# Enhancing Confusion Entropy (CEN) for binary and multiclass classification

**DOI:** 10.1371/journal.pone.0210264

**Published:** 2019-01-14

**Authors:** Rosario Delgado, J. David Núñez-González

**Affiliations:** 1 Department of Mathematics, Universitat Autònoma de Barcelona, Campus de la UAB, Cerdanyola del Vallès, Spain; 2 Department of Mathematics, University of the Basque Country (UPV/EHU), Leioa, Spain; Universita degli Studi di Palermo Dipartimento di Fisica e Chimica, ITALY

## Abstract

Different performance measures are used to assess the behaviour, and to carry out the comparison, of classifiers in Machine Learning. Many measures have been defined on the literature, and among them, a measure inspired by Shannon’s entropy named the Confusion Entropy (CEN). In this work we introduce a new measure, MCEN, by modifying CEN to avoid its unwanted behaviour in the binary case, that disables it as a suitable performance measure in classification. We compare MCEN with CEN and other performance measures, presenting analytical results in some particularly interesting cases, as well as some heuristic computational experimentation.

## Introduction

Machine Learning is the subfield of Computer Science, as well as the branch of Artificial Intelligence, whose objective is to develop techniques that allow computers to learn. It has a wide range of applications, such as search engines or pattern recognition. Examples are: medical diagnosis, fraud detection, stock market analysis, classification of DNA sequences, recognition of speech and written language, images, games and robotics.

Machine learning tasks are typically grouped into two broad categories: Supervised and Unsupervised Learning. Classification falls in the former, since it deals with some input variables (features or characteristics) and an output variable (the class), and uses an algorithm to infer the class of (that is, to classify) a new case from its known features. Different models are used to build classifiers. Decision Trees (J48, Random Forest), Rules (Decision Table, JRip, ZeroR), Neural Networks (Multilayer Perceptron, Extreme Learning Machines, RBFN), Support Vector Machines, and Bayesian Networks (Naive Bayes, TAN) are some, although not the only ones, approximations to supervised classification.

Once a classifier is built, a performance measure is needed in order to assess its behaviour and to compare it with other classifiers. In the binary case, in which the class variable has only two labels or classes, there are several classical measures that have been widely used: Accuracy, Sensitivity, Specificity and F-score, only to mention some of the most commonly used. Not of all them allow a natural extension to the multi-class case (more than two labels), and only few measures have been specially designed for multi-class classification, which is a more complex scenario. Accuracy, by far the simplest and widespread performance measure in classification, extends seamlessly its definition in the binary case to multi-class classification. Another well known performance measure, formerly introduced in the binary case but that extends without problems, is Matthew’s Correlation Coefficient (MCC), introduced by Matthews in [1].

In this work, whose seed is [2], we focus on a different performance measure, named Confusion Entropy (CEN), which measures the uncertainty generated by classification, and has been recently introduced by Wang et al. in [3] as a novel measure for evaluating classifiers based on the concept of Shannon’s entropy. CEN measures generated entropy from misclassified cases considering not only how the cases of each fixed class have been misclassified into other classes, but also how the cases of the other classes have been misclassified as belonging to this class, as well as entropy inside well-classified cases. Given a set of non-negative numbers, say {*n*_1_, …, *n*_*r*_}, the Shannon’s entropy generated by the set can be defined as the sum $\sum_{i=1}^{r}-p_{i}\;\log\left(p_{i}\right)$, with $p_{i}=\frac{n_{i}}{n}$ if $n=\sum_{i=1}^{r}n_{i}$, where log can be, as usual, the logarithm in base 2.

CEN is compared in [3] with Accuracy and other measures, showing a relative consistency with them: higher Accuracy tends to result in lower Confusion Entropy. This performance measure, which is more discriminating for evaluating classifiers than Accuracy, specially when the number or cases grows, has also been studied in [4], where the authors show the strong monotone relation between CEN and MCC, and that both, MCC and CEN, improve over Accuracy.

There are some works in the recent literature using Confusion Entropy. For example, in [5] the authors propose a novel splitting criterion based on CEN for learning decision trees with higher performance; experimental results on some data sets show that this criterion leads to trees with better CEN value without reducing accuracy. The authors of [6] and [7] use CEN, among other performance measures, to compare several common data mining methods used with highly imbalanced data sets where the class of interest is rare. Other works propose modifications of this measure, as [8], in which a Confusion Entropy measure based on a probabilistic confusion matrix is introduced, measuring if cases are classified into true classes and separated from others with high probabilities. A similar approach to that of [8] is followed in [9] to analyze the probability sensitivity of the Gaussian processes in a bankruptcy prediction context, by means of a probabilistic confusion entropy matrix based on the model estimated probabilities. In the context of horizontal collaboration, the system global entropy is introduced in [10] analogously to CEN (see also [11] and [12]), and it is used in the collaborative part of a clustering algorithm, which is iterative with the optimization process continuing as long as the system global entropy is not stable.

It is remarkable that CEN shows to have a weakness in the binary case that invalidates it as a suitable performance measure: in some situations CEN gets values larger than one, unlike what happens in the multi-class case, in which CEN ranges between zero and one. CEN is a measure of the “overall” entropy associated to the confusion matrix, that can be thought as generated by two sources: entropy within the main diagonal, and the one generated by the values outside it, corresponding to misclassification. We will show that CEN is more sensible to the later. A second but not least important point in the weakness of the behaviour of CEN is its lack of monotonicity when the overall entropy does increase (or decrease) monotonously. Along the paper we will show different situations to stand out these items.

Our aim is to introduce an enhanced CEN measure, that we denote by MCEN, and compare it with CEN, MCC and Accuracy. This new measure will show to be highly correlated with CEN. Two aspects deserve to be highlighted:

1. definitions of probabilities involved in the construction of CEN have been modified in MCEN to improve interpretability as real probabilities,
2. weakness of CEN in the binary case (out-of-range and lack of monotonicity) are overcome with MCEN.

The paper is structured as follows: first we introduce the Modified Confusion Entropy MCEN and deal with the multi-dimensional perfectly symmetric and balanced case, which is deeply studied, performing a cross comparison between CEN, MCEN, Accuracy and MCC. The general binary case is treated next, focusing on different families of matrices and carrying out the corresponding cross comparisons. Next part is devoted to study the *Z*_*A*_ family of confusion matrices. Then, we compare CEN, MCEN, Accuracy and MCC with two recently introduced measures: the Probabilistic Acuracy PACC ([13]) and the Entropy-Modulated Accuracy EMA ([14]). Finally, some experiments performed in the binary setting to compare CEN with MCEN through four real database sets are included in the Supporting Information file. These experiments show that their behaviour is mostly analog, but when it is not the case, MCEN is the one that behaves more according to entropy generated by misclassification. The paper finishes with a conclusion section.

## Methods

Given a multi-class classifier learned from a training dataset, with *N* ≥ 2 classes labelled {1, 2, …, *N*}, we apply it in order to classify cases from a testing dataset, that is, to infer the class of the cases from their known features or characteristics. Since for the cases in the testing dataset we actually know the class to which they belong, we can construct the *N* × *N* confusion matrix *C* = (*C*_*i*,*j*_)_*i*,*j*=1, …, *N*_, which collects the results issued by the classifier over the testing dataset. *C*_*i*,*j*_ is the number of cases of class *i* that have been classified as belonging to class *j*. We denote by *S* the sum of values of the matrix, that is, the total number of cases in the testing dataset, $S=\sum_{i=1}^{N}\sum_{j=1}^{N}C_{i,j}$.

We introduce notations OUT(*C*) and IN(*C*), respectively, to denote the Shannon’s entropy generated by the elements of outside (respectively, inside) the main diagonal of matrix *C*. That is, while IN is the entropy generated by the well classified cases, OUT is generated by misclassification.

In [3] the misclassification probability of classifying class-*i* cases as being of class *j* “subject to class *j*”, denoted by $P_{i,j}^{j}$, is introduced as:

$$\begin{array}{c} P_{i,j}^{j}=\frac{C_{i,j}}{\sum_{k=1}^{N}(C_{j,k}+C_{k,j})},\;\;i,j=1,...,N,\;i\neq j\;, \end{array}$$ (1)

that is, $P_{i,j}^{j}$ is “almost” the relative frequency class-*i* cases that are classified as being of class *j* among all cases that are of class *j* or that have been classified as being of class *j*. But not exactly. The reason is that class-*j* cases that have been correctly classified, whose number is *C*_*j*,*j*_, are counted twice in the denominator.

Analogously, the misclassification probability of classifying class-*i* cases as being of class-*j* “subject to class *i*”, with analogous interpretation, denoted by $P_{i,j}^{i}$, is defined in the same paper by:

$$\begin{array}{c} P_{i,j}^{i}=\frac{C_{i,j}}{\sum_{k=1}^{N}(C_{i,k}+C_{k,i})},\;\;i,j=1,...,N,\;i\neq j\;. \end{array}$$ (2)

Then, the Confusion Entropy associated to class *j* is defined in [3] by:

$$\begin{array}{c} {\text{CEN}}_{j}=-\sum_{k=1,k\neq j}^{N}(P_{j,k}^{j}\log_{2(N-1)}\left(P_{j,k}^{j}\right)+P_{k,j}^{j}\log_{2(N-1)}\left(P_{k,j}^{j}\right)) \end{array}$$ (3)

with the convention *a* log_*b*_(*a*) = 0 if *a* = 0. Finally, the overall Confusion Entropy associated to the confusion matrix *C* is defined as a convex combination of the Confusion Entropy of the classes as follows:

$$\begin{array}{c} \text{CEN}=\sum_{j=1}^{N}P_{j}\;{\text{CEN}}_{j}\;, \end{array}$$ (4)

where the non-negative weights *P*_*j*_, summing 1, are

$$\begin{array}{c} P_{j}=\frac{\sum_{k=1}^{N}(C_{j,k}+C_{k,j})}{2\sum_{k,\ell =1}^{N}C_{k,\ell }}\;. \end{array}$$ (5)

Note that CEN is an invariant measure; if we multiply all elements of the confusion matrix by a constant we obtain the same result. The same convenient and useful property holds with Accuracy, MCC and the modified Confusion Entropy measure MCEN, that we will introduce below. As MCC lives in [−1, 1] while Accuracy, CEN and MCEN range in [0, 1], we scale MCC and introduce ${\text{MCC}}^{*}=\frac{1-\text{MCC}}{2}\in \left[0,\;1\right]$. Besides, since Accuracy usually has an inverse relationship with both CEN and MCEN, we choose to consider ACC* = 1–Accuracy instead of Accuracy itself.

For *N* > 2, CEN ranges between 0 and 1, 0 is attained with perfect classification (the off-diagonal elements of matrix *C* being zero), while 1 under complete misclassification, symmetry and balance in *C*, that is, if all diagonal elements in *C* are zero, and the off-diagonal elements take all the same value. In the binary case (*N* = 2), although CEN remains to be 0 with perfect classification, and is 1 under complete misclassification with symmetry, in intermediate scenarios we can also obtain CEN = 1 and even higher values. That is, in some cases CEN is out-of-range. See, for example, the confusion matrices in Table 1, which have already been considered in [4]. The lack of monotonicity when the situation monotonously goes from perfect classification to completely symmetric and balanced misclassification, as showed by the sequence of matrices in Table 1, represents a great inconvenience of CEN in the binary case, and is our main motivation for introducing a modified version of it.

**Table 1:** Examples in the perfectly symmetric and balanced binary case with *S* = 12. Only CEN values.

	$\left(\begin{array}{cc} 6 & 0\\ 0 & 6 \end{array}\right)$	$\left(\begin{array}{cc} 5 & 1\\ 1 & 5 \end{array}\right)$	$\left(\begin{array}{cc} 4 & 2\\ 2 & 4 \end{array}\right)$	$\left(\begin{array}{cc} 3 & 3\\ 3 & 3 \end{array}\right)$	$\left(\begin{array}{cc} 2 & 4\\ 4 & 2 \end{array}\right)$	$\left(\begin{array}{cc} 1 & 5\\ 5 & 1 \end{array}\right)$	$\left(\begin{array}{cc} 0 & 6\\ 6 & 0 \end{array}\right)$
CEN =	0.0000	0.5975	0.8617	1.0000	1.0566	1.0525	1.0000

### Definition

Instead of (1), we propose to introduce the probability of classifying class-*i* cases in class *j* “subject to class *j*”, as

$$\begin{array}{c} {\tilde{P}}_{i,j}^{j}=\frac{C_{i,j}}{\sum_{k=1}^{N}(C_{j,k}+C_{k,j})-C_{j,j}},\;\;i,j=1,...,N,\;i\neq j\;. \end{array}$$

that is, we overcome the fact that in (1) correctly classified class-*j* cases are counted twice in the denominator. With this definition, ${\tilde{P}}_{i,j}^{j}$ is really the relative frequency of class-*i* cases classified as belonging to class *j* among all cases that are of class *j* or that have been classified as being of class *j*. Analogously, we modify definition (2) in the same sense:

$$\begin{array}{c} {\tilde{P}}_{i,j}^{i}=\frac{C_{i,j}}{\sum_{k=1}^{N}(C_{i,k}+C_{k,i})-C_{i,i}},\;,i,j=1,...,N,\;i\neq j\;, \end{array}$$

and ${\tilde{P}}_{i,j}^{i}$ is really the relative frequency of class-*i* cases classified in class *j* among all cases that are of class *i* or that have been classified as being of class *i*.

Next, we modify definition of the weights in (5) in the following way:

$$\begin{array}{c} {\tilde{P}}_{j}=\frac{\sum_{k=1}^{N}(C_{j,k}+C_{k,j})-C_{j,j}}{2\;\sum_{k,\ell =1}^{N}C_{k,\ell }-\alpha \;\sum_{k=1}^{N}C_{k,k}}\;, \end{array}$$

where

$$\begin{array}{c} \alpha =\{\begin{array}{cc} 1/2 & \text{if}\;N=2\\ 1 & \text{if}\;N>2\;. \end{array} \end{array}$$

Then, we define the Confusion Entropy associated to class *j* as in (3) by

$$\begin{array}{c} {\text{MCEN}}_{j}=-\sum_{k=1,k\neq j}^{N}({\tilde{P}}_{j,k}^{j}\log_{2(N-1)}\left({\tilde{P}}_{j,k}^{j}\right)+{\tilde{P}}_{k,j}^{j}\log_{2(N-1)}\left({\tilde{P}}_{k,j}^{j}\right))\;, \end{array}$$

and the modified Confusion Entropy as in formula (4), that is,

$$\begin{array}{c} \text{MCEN}=\sum_{j=1}^{N}{\tilde{P}}_{j}\;\;{\text{MCEN}}_{j}\;. \end{array}$$ (6)

Note that when $N>2,\;\sum_{j=1}^{N}{\tilde{P}}_{j}=1$, so the modified overall Confusion Entropy is also defined as a convex combination of the modified Confusion Entropy corresponding to the classes, while in the binary case (*N* = 2), it is just defined as a conical combination since although the weights ${\tilde{P}}_{j}$ are non-negative, they do not necessarily sum up to 1 (indeed, their sum is 1 if and only if all the diagonal elements of the confusion matrix *C* are zero, that is, if all cases have been misclassified).

We see from (4) and (6) that both measures CEN and MCEN, are decomposable along classes, which makes it easy to assess the effect on the behaviour of the classifier of a simple modification affecting just one class.

We can start performing a preliminary comparison of the behaviour of ACC*, MCC*, CEN and MCEN in the toy example in dimension 2 of Table 2. In this example, the baseline confusion matrix is constant with all its entries equal to 3. First, maintaining the total sum equal to *S* = 12 and the out-diagonal invariant, we reduce the entropy IN in Table 2(a). In the baseline case, the diagonal elements are the set {3, 3}, whose entropy is 1 (maximum value). The corresponding values of IN in case (a) are consigned in Table 2, in a decreasing order. Analogously for Table 2(b) but in this case changes have been introduced outside the main diagonal. We observe that while ACC* remains insensitive to changes in the arrangement of the elements of the matrix, since the sum of the main diagonal remains constant, MCC* only decreases with decreasing entropy OUT, while when IN decreases, its value increases. As far as their interpretation is concerned, both CEN and MCEN measure the overall entropy of the confusion matrix, giving less weight to the IN entropy, that is, that generated by the well classified cases, than to OUT entropy, corresponding to misclassification. In this example we observe how their values are reduced when IN decreases, maintaining its constant sum, or when the one that is reduced is OUT, but in this second case the reduction is much more drastic, both for CEN and MCEN, and more sharply for the second. The main difference between CEN and MCEN in this sense is that the former is more sensitive to changes of IN entropy than MCEN, while less than CEN to that of OUT (observe the percentages in brackets in Table 2, which are the relative reduction in the measure with respect to that of the baseline case).

**Table 2:** Toy example: Binary case with *S* = 12. (a): Entropy reduction within the main diagonal, IN. (b) Entropy reduction outside the main diagonal, OUT. In brackets the relative reduction in each measure with respect to the baseline case. Entropy refers to IN in (a) and to OUT in (b).

	Baseline	(a)			(b)		
	$\left(\begin{array}{cc} 3 & 3\\ 3 & 3 \end{array}\right)$	$\left(\begin{array}{cc} 2 & 3\\ 3 & 4 \end{array}\right)$	$\left(\begin{array}{cc} 1 & 3\\ 3 & 5 \end{array}\right)$	$\left(\begin{array}{cc} 0 & 3\\ 3 & 6 \end{array}\right)$	$\left(\begin{array}{cc} 3 & 2\\ 4 & 3 \end{array}\right)$	$\left(\begin{array}{cc} 3 & 1\\ 5 & 3 \end{array}\right)$	$\left(\begin{array}{cc} 3 & 0\\ 6 & 3 \end{array}\right)$
Entropy =	1.0000	0.9183	0.6500	0.0000	0.9183	0.6500	0.0000
		(8.17%)	(35.00%)	(100.00%)	(8.17%)	(35.00%)	(100.00%)
ACC* =	0.5000	0.5000	0.5000	0.5000	0.5000	0.5000	0.5000
MCC* =	0.5000	0.5130	0.5625	0.6667	0.4881	0.4375	0.3333
CEN =	1.0000	0.9898	0.9575	0.8962	0.9591	0.8250	0.5000
		(1.02%)	(4.25%)	(10.38%)	(4.09%)	(17.50%)	(50.00%)
MCEN =	0.9057	0.9006	0.8848	0.8571	0.8590	0.7057	0.3343
		(0.56%)	(2.31%)	(5.37%)	(5.16%)	(22.08%)	(63.09%)

**Fig 1:**
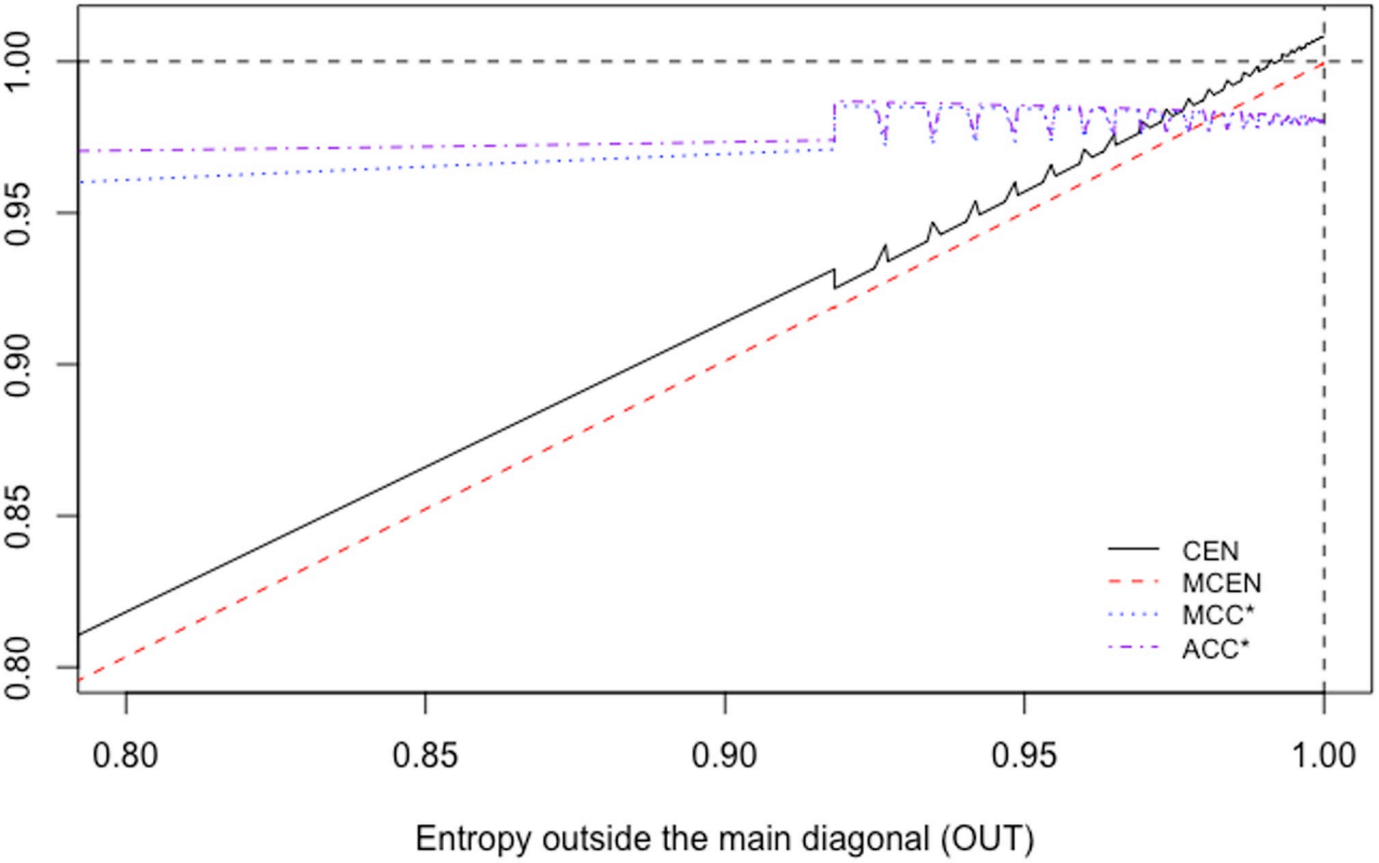
CEN, MCEN, ACC* and MCC* for matrix *M*_*A*_, as function of entropy outside the diagonal.

We can extend this comparison to matrices of type $M_{A}=(\begin{array}{cc} 1 & 50\\ A & 1 \end{array}\;)$, with *A* = 1, …, 100, for example. Their main diagonal stays constant. Fig 1 shows the behaviour of CEN, MCEN, ACC* and MCC* as OUT increases. We can observe that indeed, CEN is less correlated with this entropy than MCEN. The same can be observed from the correlations matrix given in Table 3.

**Table 3:** Correlation matrix (Pearson) for the measures of the family of matrices *M*_*A*_, *A* = 1, …, 100.

	CEN	MCEN	MCC*	ACC*	OUT
CEN	1.0000000	0.9999334	0.9229026	0.7783573	0.9999320
MCEN		1.0000000	0.9233945	0.7855300	0.9999963
MCC*			1.0000000	0.7340543	0.9241870
ACC*				1.0000000	0.7852756
OUT					1.0000000

**Fig 2:**
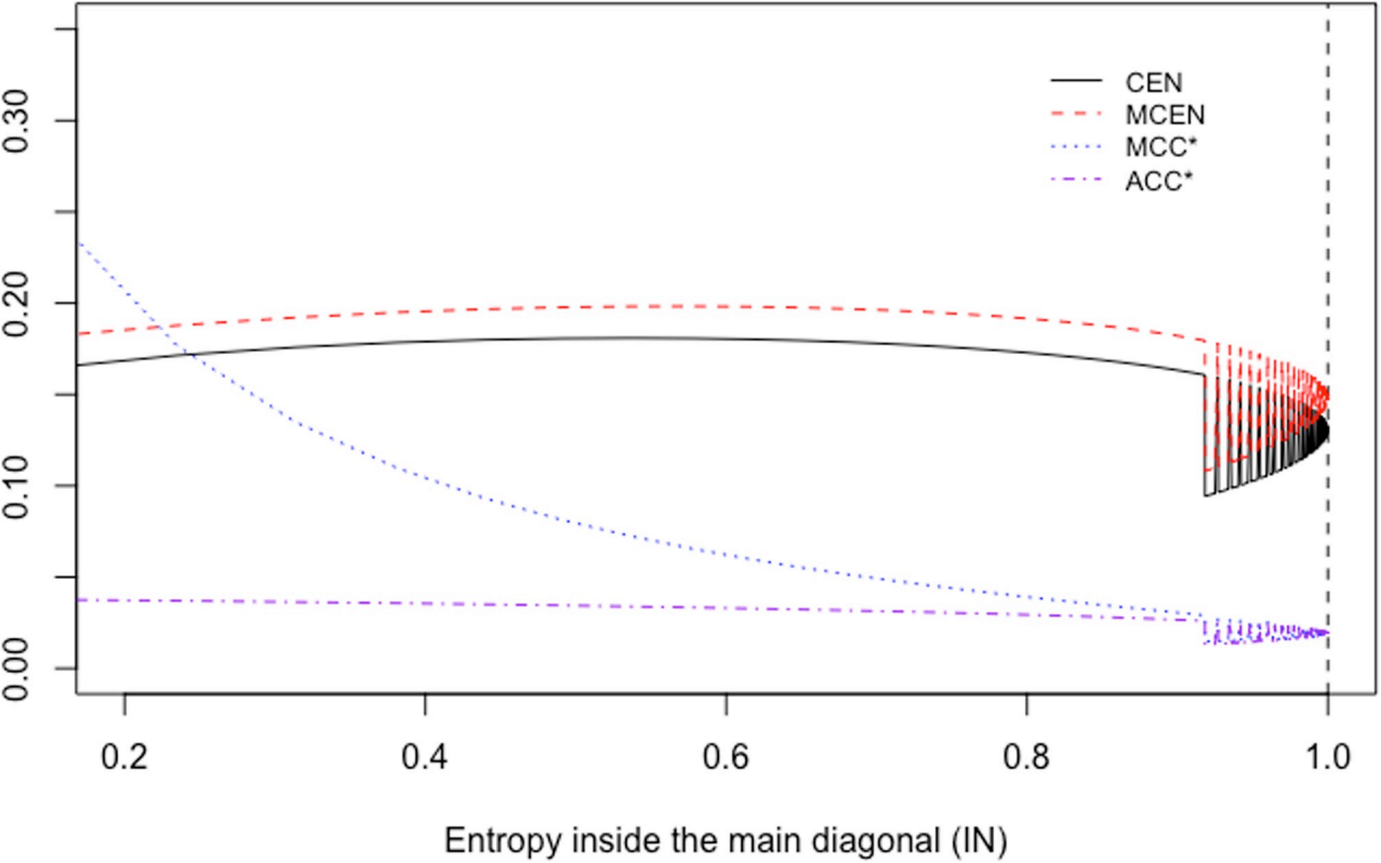
CEN, MCEN, ACC* and MCC* for matrix *W*_*A*_, as function of entropy inside the diagonal.

Instead, if we consider matrices $W_{A}=(\begin{array}{cc} 50 & 1\\ 1 & A \end{array}\;)$, with *A* = 1, …, 100, the values outside the main diagonal stay constant. Fig 2 shows the behaviour of CEN, MCEN, ACC* and MCC* as IN increases. CEN shows more correlation with this entropy than MCEN (see Table 4), although IN is less correlated (and in an inverse sense that could not be appreciated in the toy example of Table 2) than OUT, both with CEN and MCEN.

**Table 4:** Correlation matrix (Pearson) for the measures of the family of matrices *W*_*A*_, *A* = 1, …, 100.

	CEN	MCEN	MCC*	ACC*	IN
CEN	1.0000000	0.9995962	0.5499231	0.9672182	-0.6062876
MCEN		1.0000000	0.5355098	0.9609698	-0.5857654
MCC*			1.0000000	0.7340543	-0.9241870
ACC*				1.0000000	-0.7852756
IN					1.0000000

### The perfectly symmetric and balanced case

In this section we consider the case in which *C*_*i*,*j*_ = *F* for all *i*, *j* = 1, …, *N*, *i* ≠ *j* and *C*_*i*,*i*_ = *T*, with *T* ≥ 0, *F* > 0, that is, $C=(\begin{array}{ccccc} T & F & \ldots & F & F\\ F & T & \ldots & F & F\\ \vdots & \vdots & \ldots & \vdots & \vdots \\ F & F & \ldots & T & F\\ F & F & \ldots & F & T \end{array}\;)$.

**Proposition 1** *In the perfectly symmetric and balanced case*,

$$\begin{array}{cc} & If\;N>2,\;\text{CEN}=\frac{2\;(N-1)}{\delta }\;\log_{2(N-1)}\left(\delta \right),\;\text{MCEN}=\frac{2\;(N-1)}{\tilde{\delta }}\;\log_{2(N-1)}\left(\tilde{\delta }\right),\\ & If\;N=2,\;\text{CEN}=\frac{1}{1+\gamma }\;\log_{2}\left(\delta \right),\;\text{MCEN}=\frac{1}{1+\frac{3}{4}\;\gamma }\;\log_{2}\left(\tilde{\delta }\right), \end{array}$$ (7)

*where*

$$\begin{array}{c} \gamma =\frac{T}{F}\geq 0,\;\delta =2\;\left(N-1\right)+2\;\gamma >0\;and\;\tilde{\delta }=2\;\left(N-1\right)+\gamma >0\;, \end{array}$$

$$\begin{array}{c} {\text{ACC}}^{*}=\frac{N-1}{\gamma +(N-1)}\;and\;{\text{MCC}}^{*}=\frac{N}{2\;(\gamma +(N-1))}=\frac{N}{2\;(N-1)}\;{\text{ACC}}^{*}\;. \end{array}$$

Note that ACC*, MCC*, CEN and MCEN depend on the matrix values *T* and *F* only through its ratio *γ*. In (7) (case *N* > 2), CEN and MCEN have the same expression except that CEN depends on *δ*, which is function of 2*γ*, while MCEN does on $\tilde{\delta }=\delta -\gamma$, which is the same function but of *γ*. Therefore,

$$\begin{array}{c} \text{if}\;N>2,\;\text{MCEN}(2\;\gamma )=\text{CEN}(\gamma )\;, \end{array}$$

where in the notation we highlight the dependency of CEN and MCEN on *γ*.

**Corollary 1** *In the perfectly symmetric and balanced case, we have that*:

- *For any N* > 2, CEN, MCEN, ACC* *and* MCC* *are monotonically decreasing functions of γ* ≥ 0, *with*
  $$\begin{array}{cc} & \lim_{\gamma \to +\infty }\text{CEN}\left(\gamma \right)=\lim_{\gamma \to +\infty }\text{MCEN}\left(\gamma \right)=\lim_{\gamma \to +\infty }{\text{ACC}}^{*}\left(\gamma \right)=\lim_{\gamma \to +\infty }{\text{MCC}}^{*}\left(\gamma \right)=0\;,\\ & \text{CEN}\left(0\right)=\text{MCEN}\left(0\right)={\text{ACC}}^{*}\left(0\right)=1,\;\;{\text{MCC}}^{*}\left(0\right)=\frac{N}{2\;(N-1)}\to \frac{1}{2}\;as\;N\to +\infty , \end{array}$$
  *and if γ* > 0, MCC* < ACC* < CEN < MCEN.
- *Nevertheless, when N* = 2, *we have that although* MCEN *and* ACC* = MCC* *remain to be monotonically decreasing as functions of γ* ≥ 0, CEN *does not*. *Indeed*, CEN *achieves its global maximum when* $\gamma =\frac{e}{2}-1$, *which is* $\text{CEN}(\frac{e}{2}-1)\approx 1.06148>1$. *More specifically*,
  $$\begin{array}{cc} \text{CEN}(0) & =\text{CEN}\left(1\right)=1,\;\;\text{CEN}\left(\gamma \right)>1\;,\;\;\text{for}\;\text{all}\;\;\;0<\gamma <1,\;\;\lim_{\gamma \to +\infty }\text{CEN}\left(\gamma \right)=0,\\ \text{MCEN}(0) & =1\;,\;\lim_{\gamma \to +\infty }\text{MCEN}\left(\gamma \right)=0\;,\\ {\text{ACC}}^{*}\left(0\right) & ={\text{MCC}}^{*}\left(0\right)=1\;,\;\lim_{\gamma \to +\infty }{\text{ACC}}^{*}\left(\gamma \right)=\lim_{\gamma \to +\infty }{\text{MCC}}^{*}\left(\gamma \right)=0\;. \end{array}$$
  *Moreover, there exists γ*_0_ ≈ 5.78 *such that*
  $$\begin{array}{cc} {\text{MCC}}^{*} & ={\text{ACC}}^{*}<\text{MCEN}<\text{CEN}\;\;\;if\;\;\;0<\gamma <\gamma_{0},\\ {\text{MCC}}^{*} & ={\text{ACC}}^{*}<\text{MCEN}=\text{CEN}\;\;\;if\;\;\;\gamma =\gamma_{0},\;\text{and}\\ {\text{MCC}}^{*} & ={\text{ACC}}^{*}<\text{CEN}<\text{MCEN}\;\;\;if\;\;\;\gamma >\gamma_{0}\;. \end{array}$$

**Proof 1** *The proofs of both Proposition 1 and Corollary 1 are straightforward, and then omitted. However, it is worth mentioning that in order to prove* CEN < MCEN *in case N* > 2 *we use that function* $f\left(x\right)=\frac{1}{x}\;\log_{b}\left(x\right)$ *is strictly decreasing for any base b* > 1 *(in our case, b* = 2(*N* − 1) ≥ 4), *and x* > *e*. *We apply that fact to see that f*(*x*_0_) > *f*(*x*_1_) *with* *x*_0_ = 2(*N* − 1) + *γ* < *x*_1_ = 2(*N* − 1) + 2*γ*, *since* *x*_0_ ≥ 4 > *e*.

*The same property of function f allows to prove that both* CEN *and MCEN are monotonically decreasing as functions of γ, with x* = *δ* = 2(*N* − 1) + 2*γ* *and* $x=\tilde{\delta }=2\;\left(N-1\right)+\gamma$, *respectively, being both* > *e for any γ* ≥ 0. *Note that since for N* = 2 *the expression of* CEN *as function of δ is as in case N* > 2, *the monotonous decrease fails since x* = *δ* = 2 + 2*γ* < *e* *for* $\gamma <\frac{e}{2}-1$.

The rest of proofs are also omitted.

**Fig 3:**
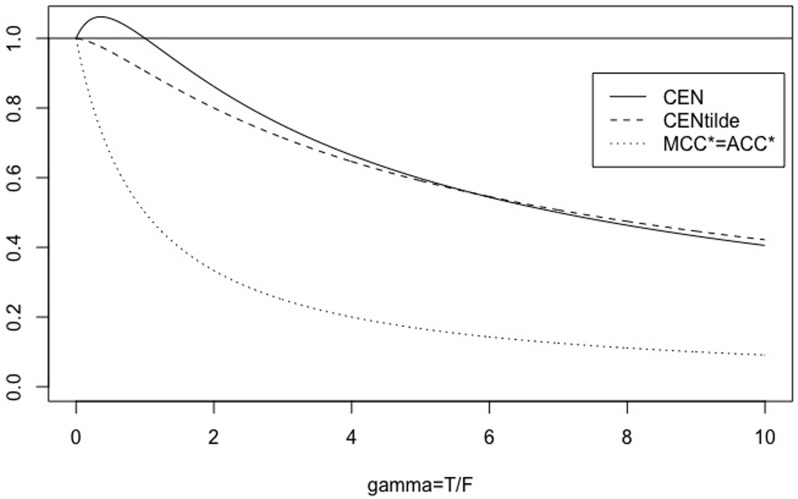
The symmetric case. CEN, MCEN, ACC* and MCC* for *γ* ∈ [0, 10], with *N* = 2.

**Remark 1** *Note that if N* = 2, CEN *exhibits the unwanted behaviour, not showed by* MCEN, *of being out-of-range* [0, 1], *which despairs for N* > 2 *(see* Figs 3 *and* 4).

**Fig 4:**
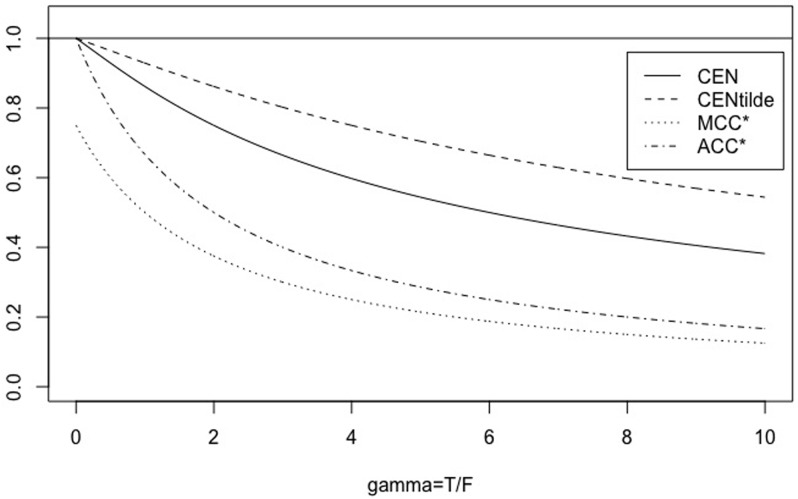
The symmetric case. CEN, MCEN, ACC* and MCC* for *γ* ∈ [0, 10], with *N* = 3.

**Remark 2** *Consider the particular case in which T* = *F, that is, γ* = 1. *In other words, the confusion matrix is constant, say* $\left(\begin{array}{cccc} 1 & 1 & \ldots & 1\\ \vdots & \vdots & \ldots & \vdots \\ 1 & 1 & \ldots & 1 \end{array}\;\right)$. *Then*, $\text{ACC}*=\frac{N-1}{N}$ *and* $\text{MCC}*=\frac{1}{2}$. *Moreover, δ* = 2*N and* $\tilde{\delta }=2\;N-1$.

*If N* > 2, $\text{CEN}=\left(1-\frac{1}{N}\right)\log_{2(N-1)}\left(2N\right)$ *and* $\text{MCEN}=\left(1-\frac{1}{2N-1}\right)\log_{2(N-1)}\left(2N-1\right).$

*If N* = 2, CEN = 1 *and* $\text{MCEN}=\frac{4}{7}\;\log_{2}\left(3\right)<1\;.$

*As a consequence, we can easily check that if N* > 2, MCC* < ACC* < CEN < MCEN, *with* lim_*N*→+∞_ ACC* = lim_*N*→+∞_ CEN = lim_*N*→+∞_ MCEN = 1, *while if N* = 2, MCC* = ACC* < MCEN < CEN.

The particular pathological case of matrices *Z*_*A*_ will be studied in the multi-class setting, but before we consider in some detail the binary case.

### The general binary case

The binary case (*N* = 2) can be studied in more detail. We will use the following notation for the confusion matrix in the most general setting, taking class 1 as reference:

$$\begin{array}{c} C=(\begin{array}{cc} TP\;\; & FN\\ FP\;\; & TN \end{array})\;, \end{array}$$ (8)

where *TP* is the true positive or number of class-1 cases that have been correctly classified, and the same for the true negative number of cases *TN* with class 2. On the other hand, *FP* denotes false positives or number of class-2 cases that have been miscllassified, and *FN* false negatives.

**Proposition 2** *If the confusion matrix C is given by* (8), *we have that with S* = *TP* + *TN* + *FP* + *FN*,

$$\begin{array}{cc} \text{CEN} & =\frac{\left(FN+FP\right)\;\log_{2}(S^{2}-{\left(TP-TN\right)}^{2})}{2\;S}-\frac{FN\;\log_{2}\left(FN\right)+FP\;\log_{2}\left(FP\right)}{S}\;,\\ \text{MCEN} & =\frac{2\left(FN+FP\right)\log_{2}((S-TN)(S-TP))}{3S+(FN+FP)}-\frac{4(FN\log_{2}\left(FN\right)+FP\log_{2}\left(FP\right))}{3S+(FN+FP)},\\ {\text{ACC}}^{*} & =\frac{FP+FN}{S}\;\;\text{and}\;\;\;{\text{MCC}}^{*}=\frac{1-\text{MCC}}{2},\\ \text{with}\;\;\; & \text{MCC}=\frac{TP\;TN-FP\;FN}{\sqrt{\left(TP+FN)\;(FP+TN)\;(TP+FP)\;(TN+FN\right)}}\;. \end{array}$$ (9)

To carry out a deeper study, we have to consider particular situations; is what we do in the subsections below, where different particular scenarios have been introduced and developed.

#### The perfectly symmetric and balanced case

Table 5 below shows some examples of 2 × 2 confusion matrices of type $\left(\begin{array}{cc} T & F\\ F & T \end{array}\right)$, that is, in which *TP* = *TN* = *T* and *FP* = *FN* = *F*. All of them correspond to *S* = 12 and have already been considered in [4]. This is a particular case of the previously considered setting, and Proposition 1 and Corollary 1 apply here. We can observe again the anomalous behaviour of CEN, in contrast with the other measures.

**Table 5:** Examples in the perfectly symmetric and balanced binary case with *S* = 12.

	$\left(\begin{array}{cc} 6 & 0\\ 0 & 6 \end{array}\right)$	$\left(\begin{array}{cc} 5 & 1\\ 1 & 5 \end{array}\right)$	$\left(\begin{array}{cc} 4 & 2\\ 2 & 4 \end{array}\right)$	$\left(\begin{array}{cc} 3 & 3\\ 3 & 3 \end{array}\right)$	$\left(\begin{array}{cc} 2 & 4\\ 4 & 2 \end{array}\right)$	$\left(\begin{array}{cc} 1 & 5\\ 5 & 1 \end{array}\right)$	$\left(\begin{array}{cc} 0 & 6\\ 6 & 0 \end{array}\right)$
ACC* = MCC* =	0.0000	0.1667	0.3333	0.5000	0.6667	0.8333	1.0000
CEN =	0.0000	0.5975	0.8617	1.0000	1.0566	1.0525	1.0000
MCEN =	0.0000	0.5910	0.8000	0.9057	0.9614	0.9891	1.0000

#### The symmetric but unbalanced family *U*_*A*_

**Fig 5:**
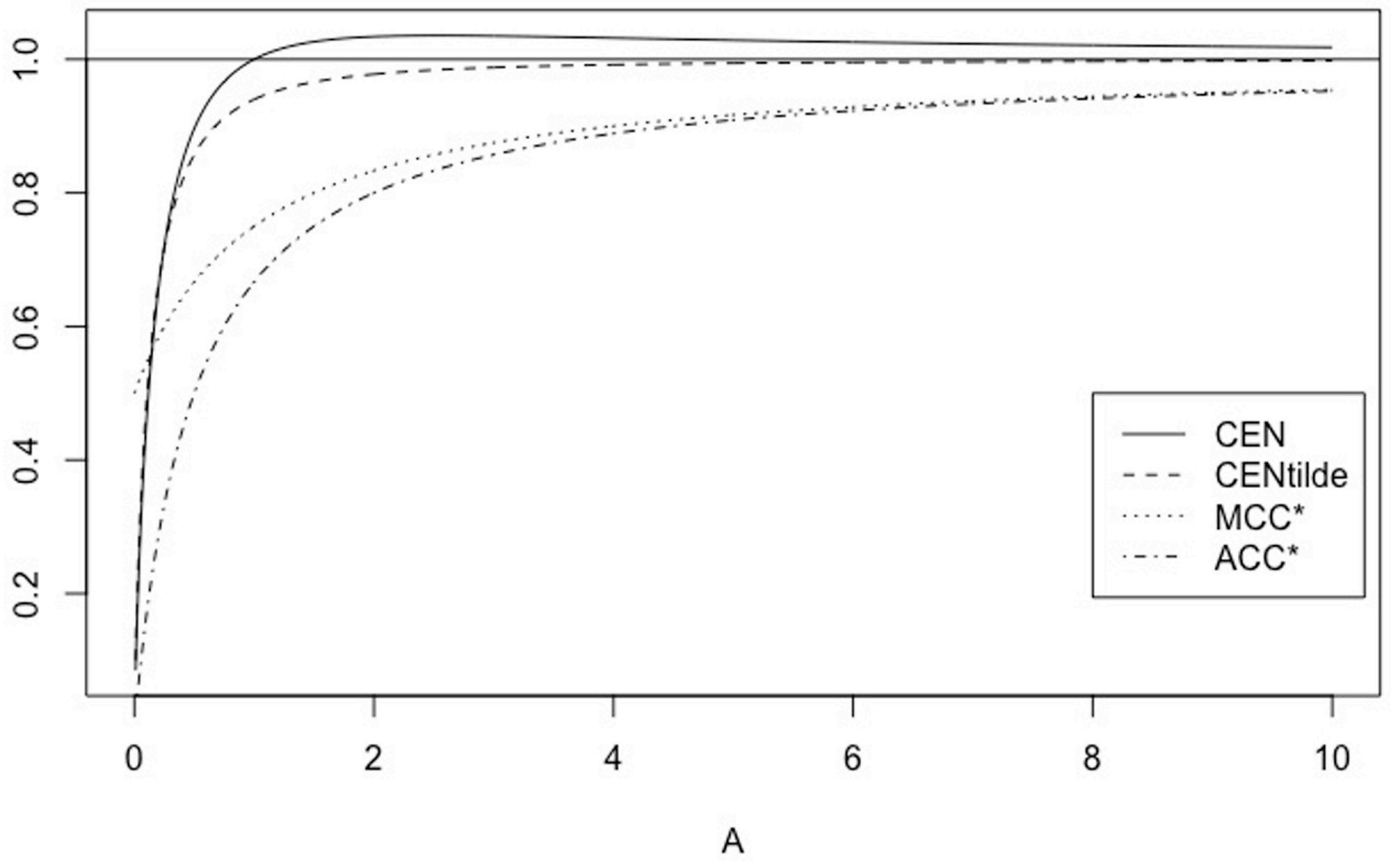
Famlily *U*_*A*_. CEN, MCEN, ACC* and MCC* for *A* ∈ (0, 10].

Consider the particular case of a confusion matrix of type $U_{A}=(\begin{array}{cc} 1 & A\\ A & 0 \end{array})$, with *A* > 0. Both class-1 and class-2 cases are mainly misclassified if *A* > 1. Entropy out of the main diagonal is 1 and within the diagonal is 0, regardless of the value of *A*. When 0 < *A* < 1, say for example that *A* = 1/*B* with *B* > 1, then matrix *U*_*A*_ is equivalent to $\left(\begin{array}{cc} B & 1\\ 1 & 0 \end{array}\right)$, that is, corresponds to an unbalanced scenario in which class 2 is underrepresented and class-1 cases are mainly well classified. We can observe some properties of CEN, MCEN, ACC* and MCC* (see Fig 5) in Proposition 3, which is derived from Proposition 2.

**Proposition 3** *For confusion matrix U*_*A*_ *with A* > 0, *we have*:

$$\begin{array}{cc} \text{CEN}(A) & =\frac{A\;\log_{2}({\left(2\;A+1\right)}^{2}-1)-2\;A\;\log_{2}\left(A\right)}{2\;A+1}\;,\\ \text{MCEN}(A) & =\frac{4\;A\;\log_{2}(2\;A\;(2\;A+1))-8\;A\;\log_{2}\left(A\right)}{3\;(2\;A+1)+2\;A}\;,\\ {\text{ACC}}^{*}\left(A\right) & =\frac{2\;A}{2\;A+1},\;{\text{MCC}}^{*}\left(A\right)=\frac{2\;A+1}{2\;(A+1)}\;. \end{array}$$

*As a consequence*:

CEN(*A*) < 1 *if A* < 1, CEN(1) = 1, CEN(*A*) > 1 *if A* > 1, MCEN(*A*) < 1 *and* ACC*(*A*) < MCC*(*A*) < 1, *for all A* > 0, MCEN, ACC* *and* MCC* *are monotonically increasing functions of A* > 0, CEN *is not, and achieves its global maximum when A* ≈ 2.54, *which is* > 1, $\underset{A\to 0}{\text{lim}}\text{CEN}(A)=\underset{A\to 0}{\text{lim}}\text{MCEN}(A)=\underset{A\to 0}{\text{lim}}{\text{ACC}}^{*}(A)=0<\underset{A\to 0}{\text{lim}}{\text{MCC}}^{*}(A)=0.5$, $\underset{A\to +\infty }{\text{lim}}\text{CEN}(A)=\underset{A\to +\infty }{\text{lim}}\text{MCEN}(A)=\underset{A\to +\infty }{\text{lim}}{\text{ACC}}^{*}(A)=\underset{A\to +\infty }{\text{lim}}{\text{MCC}}^{*}(A)=1.$

*Moreover, there exists A*_0_ ∈ (0, 1) *(indeed, A*_0_ ≈ 0.24*) such that*

$$\begin{array}{cc} & \;\text{MCEN}\left(A\right)<\text{CEN}\left(A\right)\;\;\;if\;\;\;A>A_{0},\\ & \;\text{MCEN}\left(A_{0}\right)=\text{CEN}\left(A_{0}\right),\\ & \;\text{MCEN}\left(A\right)>\text{CEN}\left(A\right)\;\;\;if\;\;\;0<A<A_{0}. \end{array}$$

The overall entropy associated to the four elements of the confusion matrix, which results to be $-\frac{2\;A}{2\;A+1}\;\log\left(\frac{A}{2\;A+1}\right)$, increases to 1 when *A* → +∞ and decreases to 0 when *A* → 0, and both CEN and MCEN, are sensible to this fact. Note that the lack of monotonicity of CEN(*A*) as *A* (and then, as the overall entropy) monotonically increases, is an anomalous behaviour that MCEN has managed to overcome. Moreover, MCEN ranges between 0 and 1. We can also observe this phenomenon in the examples in Table 6.

**Table 6:** Examples in the binary case for famlily *U*_*A*_.

	$\left(\begin{array}{cc} 10^{3} & 1\\ 1 & 0 \end{array}\right)$	$\left(\begin{array}{cc} 10^{2} & 1\\ 1 & 0 \end{array}\right)$	$\left(\begin{array}{cc} 10 & 1\\ 1 & 0 \end{array}\right)$	$\left(\begin{array}{cc} 1 & 1\\ 1 & 0 \end{array}\right)$	$\left(\begin{array}{cc} 1 & 10\\ 10 & 0 \end{array}\right)$	$\left(\begin{array}{cc} 1 & 10^{2}\\ 10^{2} & 0 \end{array}\right)$	$\left(\begin{array}{cc} 1 & 10^{3}\\ 10^{3} & 0 \end{array}\right)$
A =	1/10^3^	1/10^2^	1/10	1	10	10^2^	10^3^
ACC* =	0.00200	0.01961	0.16667	0.66667	0.952381	0.995025	0.9995002
MCC* =	0.50050	0.50495	0.54545	0.75000	0.954545	0.995050	0.9995005
CEN =	0.01194	0.08488	0.45495	1.00000	1.017859	1.002167	1.0002210
MCEN =	0.01459	0.09964	0.48263	0.93999	0.997778	0.9998483	0.9999856

#### The asymmetric family *V*_*A*_

**Fig 6:**
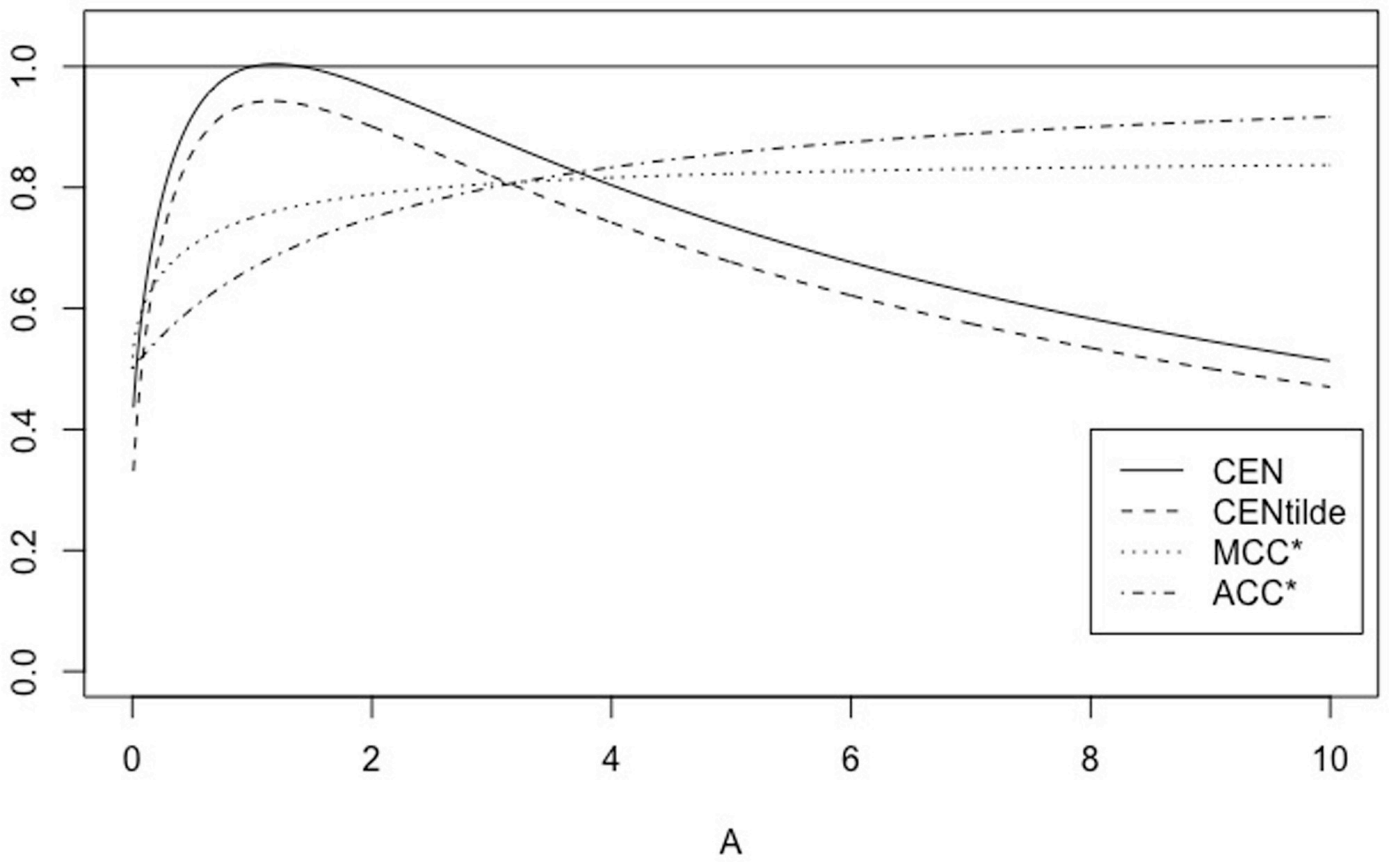
Family *V*_*A*_. CEN, MCEN, ACC* and MCC* for *A* ∈ (0, 10].

Consider the particular case of confusion matrices of type $V_{A}=(\begin{array}{cc} 1 & A\\ 1 & 0 \end{array})$, with *A* > 0. This is an asymmetric and unbalanced case in which class 2 is systematically misclassified and is underrepresented if *A* > 1. Class 1 is also mainly misclassified if *A* > 1. As *A* → +∞, entropy out the diagonal, which is $-\frac{A}{A+1}\;\log\left(\frac{A}{A+1}\right)$, decreases to zero. Entropy within diagonal is zero, while the overall entropy of the elements of matrix *V*_*A*_ is $\log\left(A+2\right)-\frac{A}{A+2}\;\log\left(A\right)$, which tends to 0 as *A* → +∞. When 0 < *A* < 1 with *A* = 1/*B*, *B* > 1, matrix *V*_*A*_ is equivalent to $\left(\begin{array}{cc} B & 1\\ B & 0 \end{array}\right)$, which corresponds to an almost balanced but asymmetric scenario in which class 1 is mainly well classified but class 2 is not. As *B* increases (*A* → 0), entropy out the diagonal also drops to zero. Some properties of CEN, MCEN, ACC* and MCC* are given in Proposition 4 (see also Fig 6).

**Proposition 4** *For confusion matrix V*_*A*_ *with A* > 0, *we have:*

$$\begin{array}{cc} \text{CEN}(A) & =\frac{\left(A+1\right)\;\log_{2}\left({\left(A+2\right)}^{2}-1\right)-2\;A\;\log_{2}\left(A\right)}{2\;(A+2)}\;,\\ \text{MCEN}(A) & =\frac{2\;\left(A+1\right)\;\log_{2}((A+1)\;(A+2))-4\;A\;\log_{2}\left(A\right)}{3\;(A+2)+(A+1)},\\ {\text{ACC}}^{*}\left(A\right) & =\frac{A+1}{A+2},\;{\text{MCC}}^{*}\left(A\right)=\frac{1+\sqrt{\frac{A}{2\;(A+1)}}}{2}\;. \end{array}$$

*As a consequence, there exists A*_1_ ∈ (1, 2) (*A*_1_ ≈ 1.414) *such that:*

CEN(*A*) > 1 *if* 1 < *A* < *A*_1_, CEN(1) = CEN(*A*_1_) = 1, CEN(*A*) < 1 *if A* ∉ [1, *A*_1_], MCEN(*A*) < 1, ACC*(*A*) < 1, MCC*(*A*) < 1 *and* MCEN(*A*) < CEN(*A*) *for all A* > 0, $\underset{A\to 0}{\text{lim}}{\text{MCC}}^{*}(A)=\underset{A\to 0}{\text{lim}}{\text{ACC}}^{*}(A)=\frac{1}{2}>\underset{A\to 0}{\text{lim}}\text{CEN}(A)=\frac{{\text{log}}_{2}(3)}{4}>\underset{A\to 0}{\text{lim}}\text{MCEN}(A)=\frac{2}{7}$, $\underset{A\to +\infty }{\text{lim}}\;{\text{ACC}}^{*}(A)\;=\;1\;>\;\underset{A\to +\infty }{\text{lim}}\;{\text{MCC}}^{*}(A)=\frac{2+\sqrt{2}}{4}\;>\;\underset{A\to +\infty }{\text{lim}}\;\text{CEN}(A)=\underset{A\to +\infty }{\text{lim}}\text{MCEN}(A)=0.$

Note that as in previous cases, CEN(*A*) does not stay always (that is, for any *A* > 0) restricted to [0, 1], while MCEN does. See Fig 6 and some examples in Table 7.

**Table 7:** Examples in the binary case for famlily *V*_*A*_.

	$\left(\begin{array}{cc} 10^{3} & 1\\ 10^{3} & 0 \end{array}\right)$	$\left(\begin{array}{cc} 10^{2} & 1\\ 10^{2} & 0 \end{array}\right)$	$\left(\begin{array}{cc} 10 & 1\\ 10 & 0 \end{array}\right)$	$\left(\begin{array}{cc} 1 & 1\\ 1 & 0 \end{array}\right)$	$\left(\begin{array}{cc} 5 & 6\\ 5 & 0 \end{array}\right)$	$\left(\begin{array}{cc} 1 & 10\\ 1 & 0 \end{array}\right)$	$\left(\begin{array}{cc} 1 & 10^{2}\\ 1 & 0 \end{array}\right)$	$\left(\begin{array}{cc} 1 & 10^{3}\\ 1 & 0 \end{array}\right)$
A =	1/10^3^	1/10^2^	1/10	1	1.2	10	10^2^	10^3^
ACC* =	0.5002	0.5025	0.5238	0.6667	0.6875	0.9167	0.9902	0.9990
MCC* =	0.5112	0.5352	0.6066	0.7500	0.7611	0.8371	0.8518	0.8535
CEN =	0.4019	0.4361	0.6217	1.0000	1.0041	0.5133	0.0934	0.0128
MCEN =	0.2921	0.3309	0.5387	0.9400	0.9429	0.4702	0.0866	0.0121

Apart from the fact that CEN is out-of-range for some values of *A*, its behaviour is similar to that of MCEN, both decreasing with entropy, while nor ACC* nor MCC* are sensitive to the decrease of entropy when *A* → +∞.

#### The symmetric but unbalanced family *X*_*A*, *r*_

Now we introduce the family of confusion matrices $X_{A,\;r}=(\begin{array}{cc} A & r\;A\\ r\;A & 1 \end{array})$, with *A*, *r* > 0. Both class-1 and class-2 cases are mainly misclassified if *A*, *r* > 1. Overall entropy of *X*_*A*, *r*_ is $-\frac{A}{(2\;r+1)\;A+1}\;\log(\frac{A}{(2\;r+1)\;A+1})-\frac{2\;r\;A}{(2\;r+1)\;A+1}\;\log(\frac{r\;A}{(2\;r+1)\;A+1})$, which drops to 0 when *A* → 0, and when *A* → +∞ converges to $\log\left(2\;r+1\right)-\frac{2\;r}{2\;r+1}\;\log\left(r\right)$, which in turn converges to 1 as *r* → +∞. Fixed *A* > 0, overall entropy converges to 1 as *r* → +∞, and as *r* → 0, it converges to $-\frac{A}{A+1}\;\log(\frac{A}{A+1})$, which in turn converges to 0 both when *A* → 0 and when *A* → +∞.

**Fig 7:**
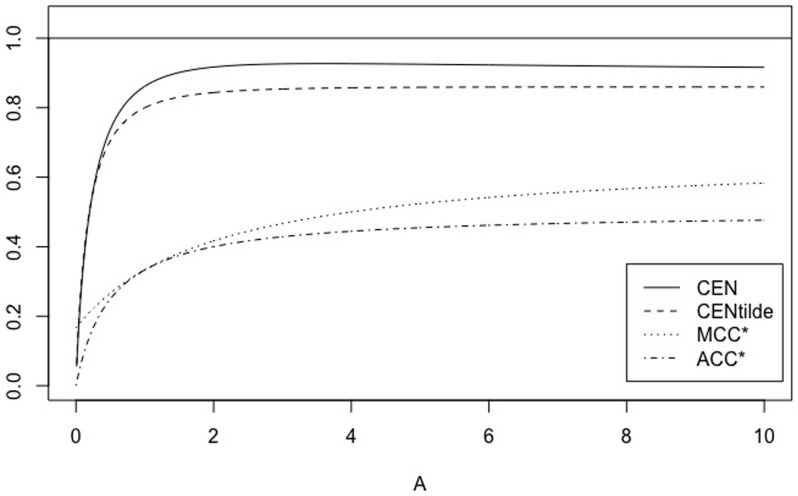
Family *X*_*A*, *r*_. CEN, MCEN, ACC* and MCC* as function of *A* > 0 for *r* = 0.5.

**Fig 8:**
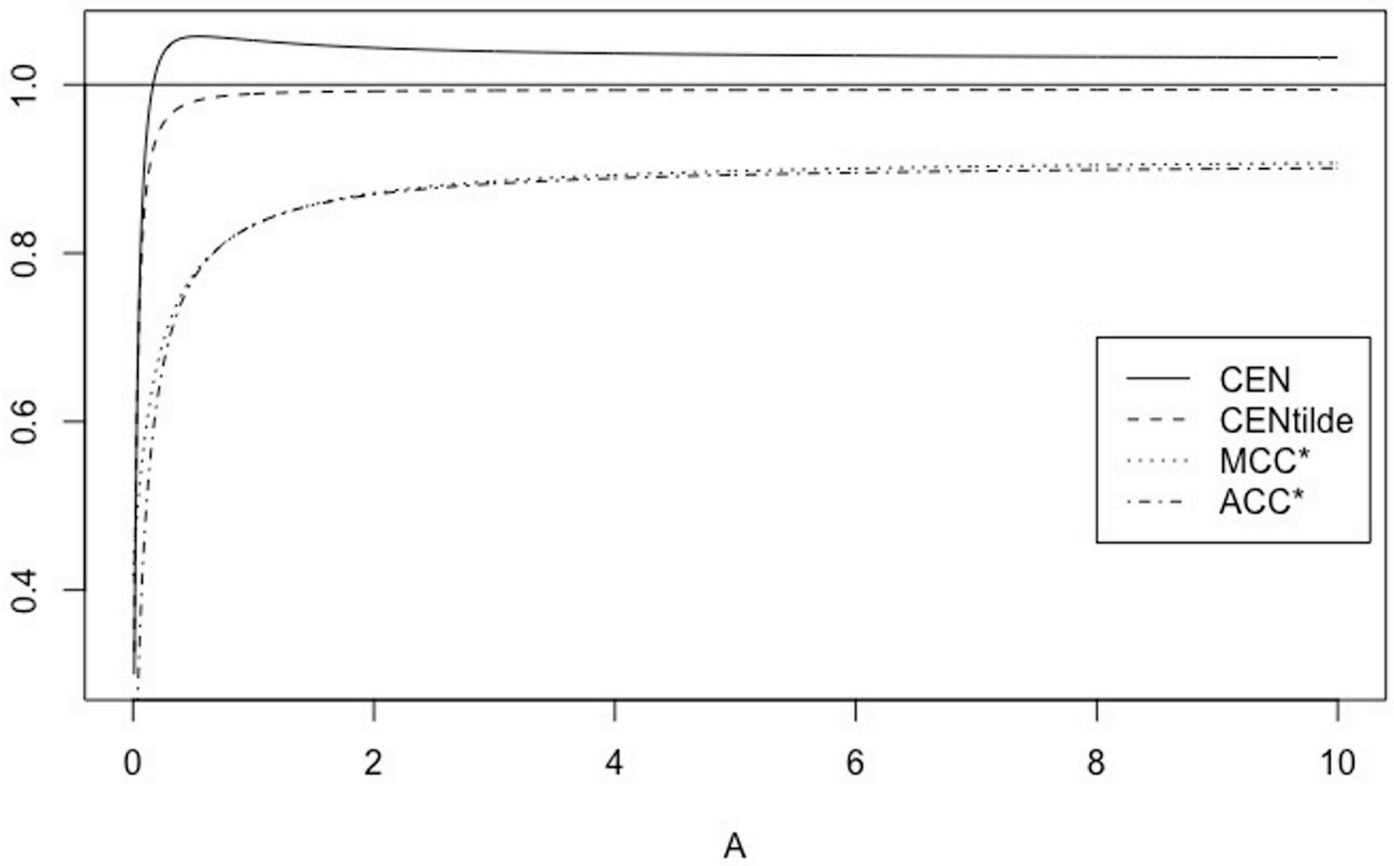
Family *X*_*A*, *r*_. CEN, MCEN, ACC* and MCC* as function of *A* > 0 for *r* = 5.

**Fig 9:**
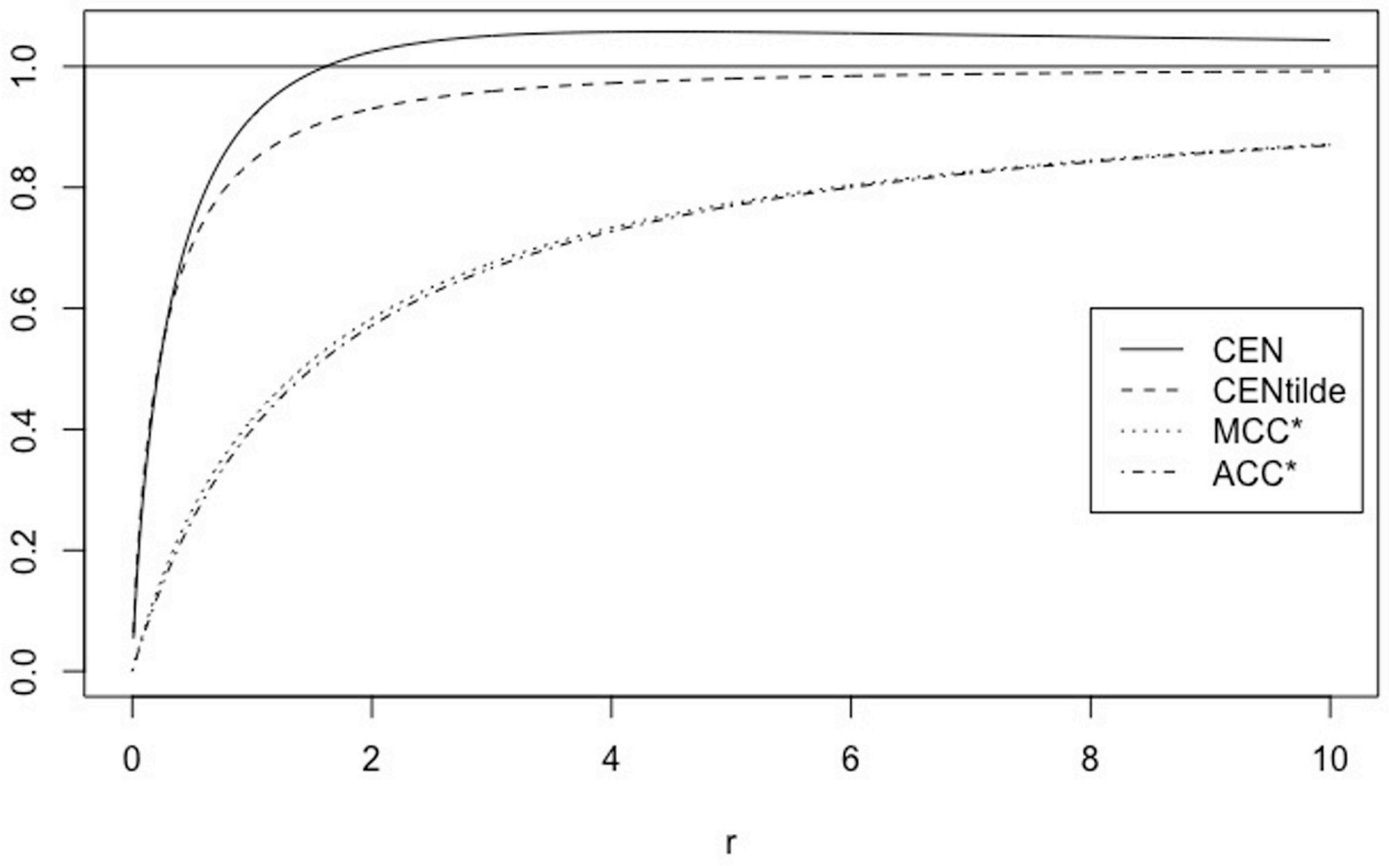
Family *X*_*A*, *r*_. CEN, MCEN, ACC* and MCC* as function of *r* > 0 for *A* = 0.5.

**Fig 10:**
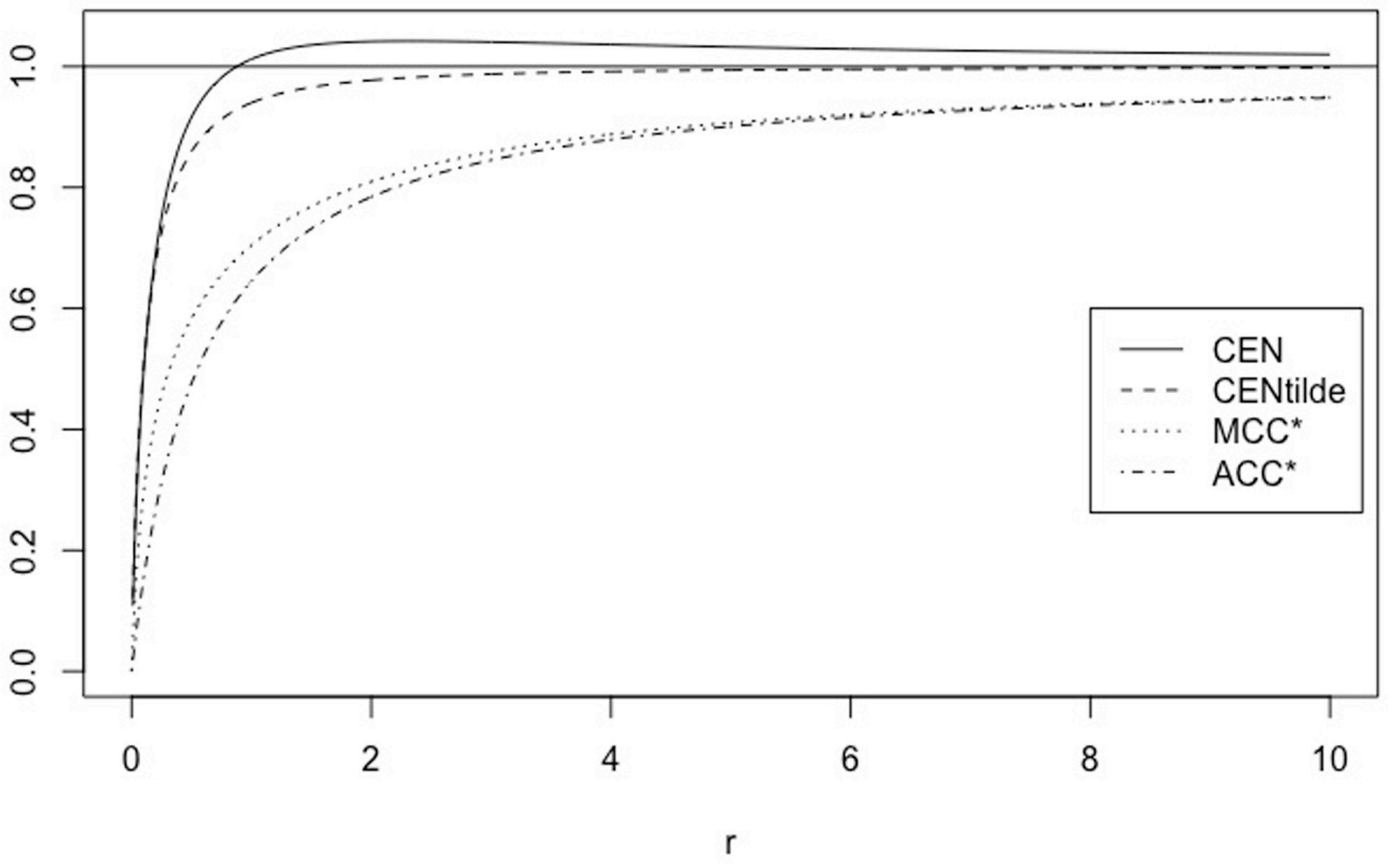
Family *X*_*A*, *r*_. CEN, MCEN, ACC* and MCC* as function of *r* > 0 for *A* = 10.

When 0 < *A* < 1, *A* = 1/*B* with *B* > 1, matrix *X*_*A*, *r*_ is equivalent to $\left(\begin{array}{cc} 1 & r\\ r & B \end{array}\right)$. We have some properties of CEN, MCEN, ACC* and MCC* in Proposition 5 below. Moreover, for *r* = 0.5, 5 Figs 7 and 8 show how the measures evolve as function of *A*, while Figs 9 and 10 show their plots as function of *r*, fixed *A* = 0.5, 10.

**Proposition 5** *For confusion matrix X*_*A*, *r*_ *with A*, *r* > 0 *we have:*

$$\begin{array}{cc} \text{CEN}(A) & =-\frac{r\;A}{(2\;r+1)\;A+1}\;\log_{2}(\frac{r^{2}\;A}{4\;(r+1)\;(r\;A+1)}),\\ \text{MCEN}(A) & =-\frac{4\;r\;A}{(8\;r+3)\;A+3}\;\log_{2}(\frac{r^{2}\;A}{\left(2\;r+1)\;(2\;r\;A+1\right)}),\\ {\text{ACC}}^{*}\left(A\right) & =\frac{2\;r\;A}{(2\;r+1)\;A+1},\;{\text{MCC}}^{*}\left(A\right)=\frac{2\;r^{2}\;A+r\;A+r}{2\;(r+1)\;(r\;A+1)}\;. \end{array}$$

*As a consequence,* $\ell_{\text{CEN}}\left(r\right)=\lim_{A\to +\infty }\text{CEN}\left(A\right)=\frac{r}{2\;r+1}\;\log_{2}(\frac{4\;(r+1)}{r})>0$, *and there exists r*_0_ < 1(*r*_0_ ≈ 0.8) *such that for any r* > *r*_0_, *there exists A*_*r*_ > 0 *such that* CEN(*A*) < 1 *if A* < *A*_*r*_, CEN (*A*_*r*_) = 1, CEN(*A*) > 1 *if A* > *A*_*r*_ *and*

$$\begin{array}{c} \ell_{\text{CEN}}\left(r\right)\{\begin{array}{cc} >1 & \;if\;r>1,\\ =1 & \;if\;r=1,\\ <1 & \;if\;r_{0}<r<1. \end{array} \end{array}$$

*If r* ≤ *r*_0_, CEN(*A*) ≤ 1 *for any A* > 0 *and ℓ*_CEN_(*r*) < 1.

*On the other hand, for any r* > 0,

MCEN(*A*) < 1, ACC*(*A*) < 1 *and* MCC*(*A*) < 1, *for all A* > 0, MCEN, ACC * *and* MCC * *are monotonically increasing functions of A*, CEN *is not, and has a global maximum, which is* > 1 *if r* > *r*_0_, $\underset{A\to 0}{\text{lim}}\text{CEN}(A)=\underset{A\to 0}{\text{lim}}\text{MCEN}(A)=\underset{A\to 0}{\text{lim}}{\text{ACC}}^{*}(A)=0,\;\underset{A\to 0}{\text{lim}}{\text{MCC}}^{*}(A)=\frac{r}{2(r+1)}$, $0<\underset{A\to +\infty }{\text{lim}}{\text{ACC}}^{*}(A)=\frac{2r}{2r+1}<\underset{A\to +\infty }{\text{lim}}{\text{MCC}}^{*}=\frac{2r+1}{2(r+1)}=l_{{\text{MCC}}^{*}}(r)<1$, $0<\underset{A\to +\infty }{\text{lim}}\text{MCEN}(A)=\frac{4r}{8r+3}{\text{log}}_{2}(\frac{2(2r+1)}{r})=l_{\text{MCEN}}(r)<1,\underset{r\to +\infty }{\text{lim}}l_{\text{MCEN}}(r)=1$.

*Moreover, there exist* 0 < *r*_3_ < *r*_2_ < *r*_1_ < *r*_0_ < 1 (*r*_3_ ≈ 0.13, *r*_2_ ≈ 0.15, *r*_1_ ≈ 0.23) *such that:*

$$\begin{array}{c} \left\{ \begin{array}{cc} \ell_{\text{MCC}}\left(r\right)>\ell_{\text{MCEN}}\left(r\right)>\ell_{\text{CEN}}\left(r\right) & \;if\;0<r<r_{3},\\ \ell_{\text{MCC}}\left(r\right)=\ell_{\text{MCEN}}\left(r\right)>\ell_{\text{CEN}}\left(r\right) & \;if\;r=r_{3},\\ \ell_{\text{MCEN}}\left(r\right)>\ell_{\text{MCC}}\left(r\right)>\ell_{\text{CEN}}\left(r\right) & \;if\;r_{3}<r<r_{2},\\ \ell_{\text{MCEN}}\left(r\right)>\ell_{\text{MCC}}\left(r\right)=\ell_{\text{CEN}}\left(r\right) & \;if\;r=r_{2},\\ \ell_{\text{MCEN}}\left(r\right)>\ell_{\text{CEN}}\left(r\right)>\ell_{\text{MCC}}\left(r\right) & \;if\;r_{2}<r<r_{1},\\ \ell_{\text{MCEN}}\left(r\right)=\ell_{\text{CEN}}\left(r\right)>\ell_{\text{MCC}}\left(r\right) & \;if\;r=r_{1},\\ \ell_{\text{CEN}}\left(r\right)>\ell_{\text{MCEN}}\left(r\right)>\ell_{\text{MCC}}\left(r\right) & \;if\;r>r_{1}. \end{array}\right. \end{array}$$

*Finally, for any fixed, A* > 0, *while MCEN*, ACC* *and* MCC* *are monotonically increasing functions of r*, CEN *is not, as can be seen in* Figs 9 *and* 10, *for two values of A*. *Given A* > 0, *there exists r*_*A*_ > *r*_0_ *such that* CEN(*A*) > 1 *for all r* > *r*_*A*_.

Note that although we do not specify it in the notations so as not to complicate them, the performance measures depend on both *A* and *r* in the case of this doubly indexed family *X*_*A*, *r*_.

#### The asymmetric family *Y*_*A*, *r*_

**Fig 11:**
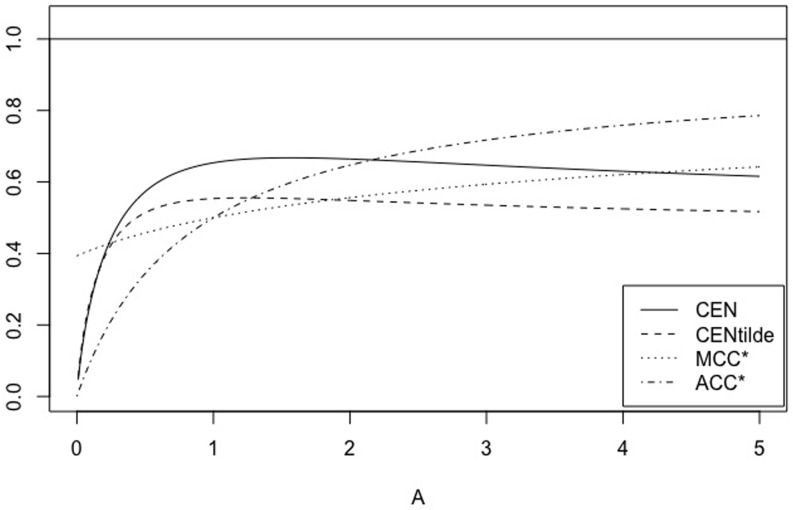
Family *Y*_*A*, *r*_. CEN, MCEN, ACC* and MCC* as function of *A* > 0 for *r* = 0.1.

**Fig 12:**
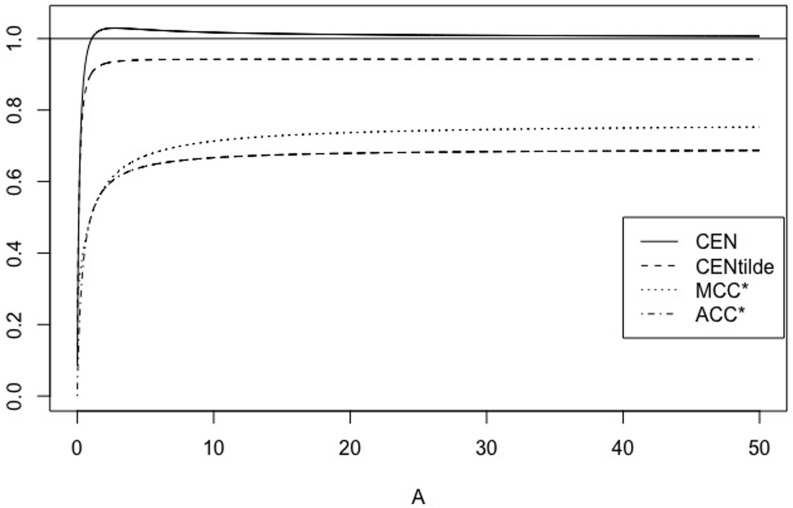
Family *Y*_*A*, *r*_. CEN, MCEN, ACC* and MCC* as function of *A* > 0 for *r* = 0.8.

**Fig 13:**
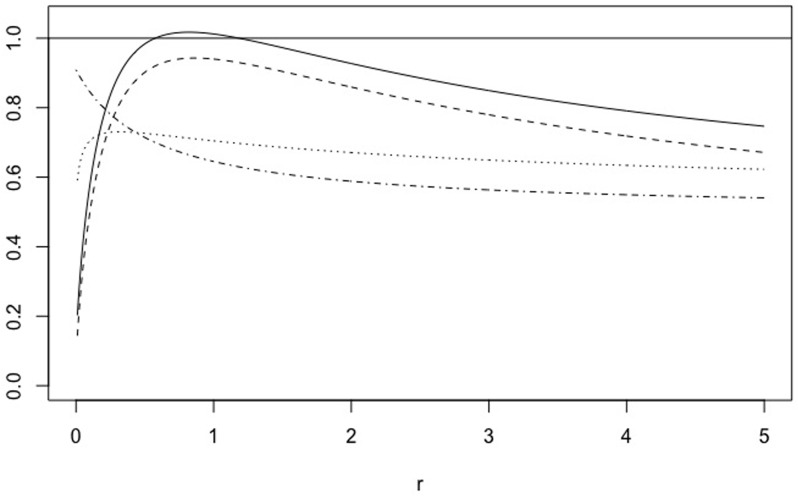
Family *Y*_*A*, *r*_. CEN, MCEN, ACC* and MCC* as function of *r* for *A* = 10.

Finally, we consider another particular doubly indexed family of confusion matrices in the binary case, with the same overall entropy as *X*_*A*, *r*_, denoted by *Y*_*A*, *r*_, with *A*, *r* > 0. We define this family by $Y_{A,\;r}=(\begin{array}{cc} r\;A & r\;A\\ A & 1 \end{array})$. Class-2 is underrepresented and mainly misclassified if *A*, *r* > 1, while class-1 cases are classified “at random”, that is, a class-1 case has the same probability to be classified into any of the two classes. Although entropy is as for *X*_*A*, *r*_, we will see that performance measures behave in a different way for this family of confusion matrices. When 0 < *A* < 1, *A* = 1/*B* with *B* > 1, then matrix *Y*_*A*, *r*_ is equivalent to $\left(\begin{array}{cc} r & r\\ 1 & B \end{array}\right)$. In Proposition 6 we give some properties of CEN, MCEN, ACC* and MCC*. See in Fig 11 for *r* = 0.1, in Fig 12 for *r* = 0.8, and see Fig 13 for a plot of them as function of *r*, fixed *A* = 10.

**Proposition 6** *For confusion matrix Y*_*A*, *r*_ *with A*, *r* > 0 *we have*:

$$\begin{array}{cc} \text{CEN}(A) & =\frac{\left(r+1\right)A\log_{2}(((r+1)A+2)\left(3r+1\right))+\left(r-1\right)A\log_{2}\left(A\right)-2rA\log_{2}\left(rA\right)}{2((2r+1)\;A+1)},\\ \text{MCEN}(A) & =\frac{2(\left(r+1\right)A\log_{2}(((r+1)A+1)\left(2r+1\right))+\left(r-1\right)A\log_{2}\left(A\right)-2rA\log_{2}\left(rA\right))}{3((2r+1)A+1)+\left(r+1\right)A},\\ {\text{ACC}}^{*}\left(A\right) & =\frac{(r+1)\;A}{(2\;r+1)\;A+1},\;{\text{MCC}}^{*}\left(A\right)=\frac{1-\frac{r\;(1-A)}{\sqrt{2\;r\;(A+1)\;(r+1)\;(r\;A+1)}}}{2}\;. \end{array}$$

*As a consequence,* $L_{\text{CEN}}\left(r\right)=\lim_{A\to +\infty }\text{CEN}\left(A\right)=\frac{1}{2\;(2\;r+1)}\;\log_{2}(\frac{{\left(\left(3\;r+1\right)\;\left(r+1\right)\right)}^{r+1}}{r^{2\;r}})>0$, *and there exists R*_0_ < 1(*R*_0_ ≈ 0.71) *such that* $L_{\text{CEN}}\left(r\right)\{\begin{array}{cc} >1 & \;if\;R_{0}<r<1,\\ =1 & \;if\;r=R_{0},\;1,\\ <1 & \;if\;r<R_{0}\;or\;r>1. \end{array}$ *Moreover, there exist* 0 < *R*_1_ < *R*_0_ < 1 < *R*_2_(*R*_1_ ≈ 0.5, *R*_2_ ≈ 1.4) *such that*

$$\begin{array}{c} \left\{ \begin{array}{cc} if\;r\in [R_{0},\;1], & \text{there}\;\text{exists}\;A_{r}>0\;\text{such}\;\text{that}\;\text{CEN}\left(A\right)<1\;if\;A<A_{r},\\ & \text{CEN}\left(A_{r}\right)=1,\;\text{CEN}\left(A\right)>1\;if\;A>A_{r},\\ if\;r\in \left(R_{1},\;R_{0}\right)\cup \left(1,\;R_{2}\right), & \;\text{there}\;\text{exist}\;0<A_{r}<B_{r}\;\text{such}\;\text{that}\;\text{CEN}\left(A\right)<1\;if\;A<A_{r}\\ & \;\text{or}\;A>B_{r},\;\text{CEN}\left(A_{r}\right)=\text{CEN}\left(B_{r}\right)=1,\\ & \text{CEN}\left(A\right)>1\;if\;A\in \left(A_{r},\;B_{r}\right),\\ \text{if}\;r\notin (R_{1},\;R_{2}), & \text{CEN}(A)\leq 1\;\text{for}\;\text{any}\;A>0. \end{array}\right. \end{array}$$

*On the other hand, for any r* > 0,

MCEN(*A*) < 1, ACC*(*A*) < 1 *and* MCC*(*A*) < 1, *for all A* > 0, ACC* *and* MCC* *are monotonically increasing functions of A*, CEN *is not, and MCEN is or not, depending on the value of r*, $\underset{A\to 0}{\text{lim}}\text{CEN}(A)=\underset{A\to 0}{\text{lim}}\text{MCEN}(A)=\underset{A\to 0}{\text{lim}}{\text{ACC}}^{*}(A)=0,\underset{A\to 0}{\text{lim}}{\text{MCC}}^{*}(A)=\frac{1-\sqrt{\frac{r}{2(r+1)}}}{2}$, $\underset{A\to +\infty }{\text{lim}}{\text{ACC}}^{*}(A)=\frac{r+1}{2r+1}=L_{{\text{ACC}}^{*}}(r),\underset{A\to +\infty }{\text{lim}}{\text{MCC}}^{*}=\frac{1+\frac{1}{\sqrt{2(r+1)}}}{2}=L_{{\text{MCC}}^{*}}(r)$, $L_{\text{MCEN}}(r)=\underset{A\to +\infty }{\text{lim}}\text{MCEN}(A)=\frac{2}{3(2r+1)+(r+1)}{\text{log}}_{2}(\frac{{\left((2r+1)(r+1)\right)}^{r+1}}{r^{2\;r}})<1,$ *L*_MCEN_(*r*) < *L*_CEN_(*r*) *for all r* > 0.

*Note that* *L*_ACC*_(*r*) < *L*_MCC*_(*r*) *if and only if* $r>\frac{-1+\sqrt{5}}{4}>0$.

#### Improving classification of the minority class while maintaining the imbalance between the classes

Up to now, we have evaluated binary confusion matrices with different balances of the two classes but not different classification results. Now let’s do just the opposite. To help clarify the utility of MCEN in the evaluation of improvements in classification of the minority class while maintaining the same amount of imbalance, we consider two different examples.

**Fig 14:**
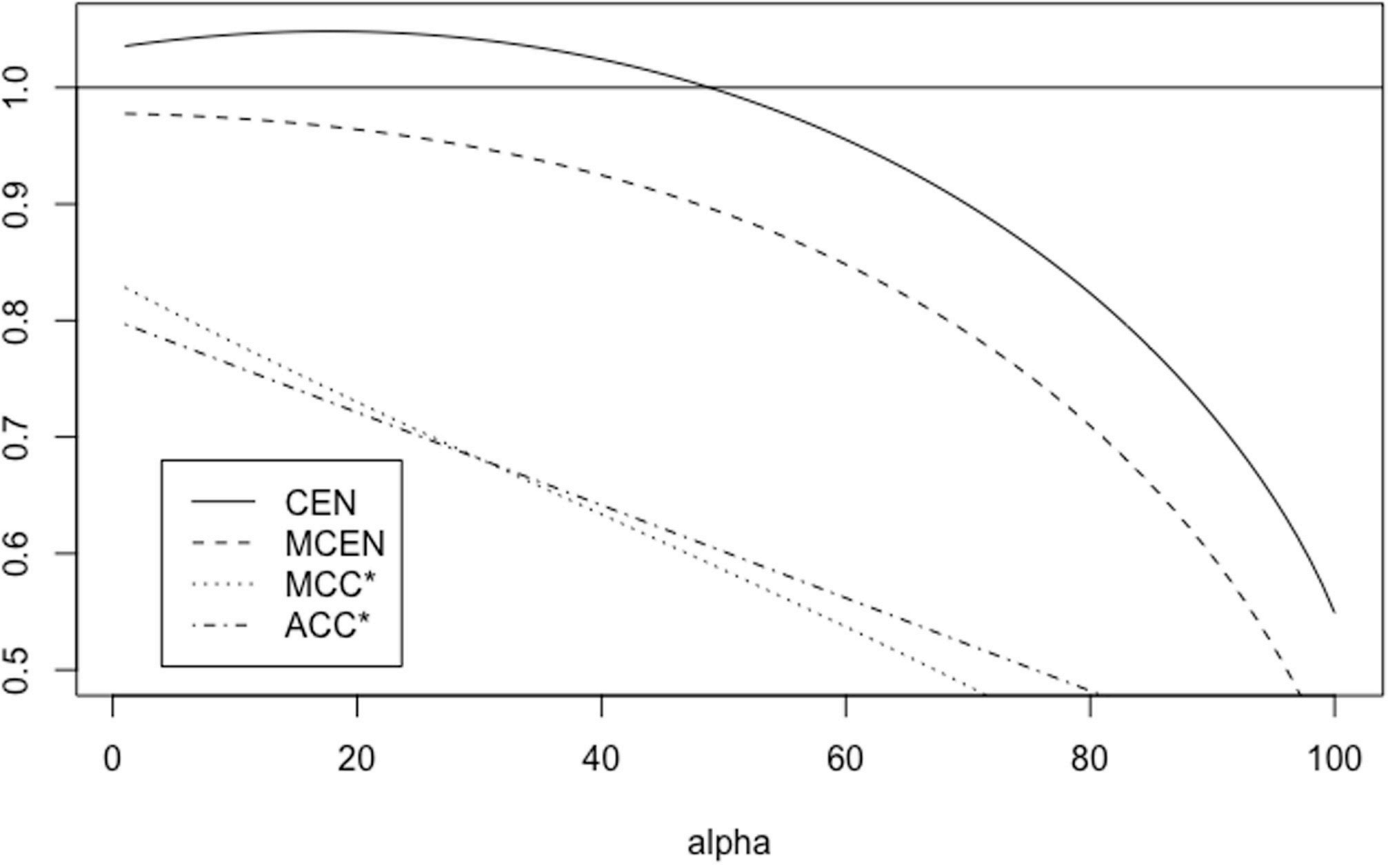
Family $X_{50,\;2}^{\alpha }$ with *α* = 1, 2, …, 101. CEN, MCEN, ACC* and MCC* as function of *α*.

**Example 1:** We introduce the family of confusion matrices $X_{50,\;2}^{\alpha }=(\begin{array}{cc} 50 & 100\\ 101-\alpha & \alpha \end{array})$, with *α* = 1, 2, …, 101. Note that when *α* = 1, the corresponding matrix belongs to the family {*X*_*A*, *r*_} with *A* = 50 and *r* = 2. Imbalance in classes stays fix. When *α* = 1, the minority class is classified very badly, improving classification as *α* increases and reaching the perfect classification when *α* = 101. Is MCEN able to detect this behaviour? Yes, it is. Unlike what happens with CEN, MCEN (as well as ACC* and MCC*) monotonically decreases when classification of the minority class improves (*α* increases). CEN incongruously first increases up to *α* = 18 and then starts to decrease and behave like the other performance measures (see Fig 14).

**Fig 15:**
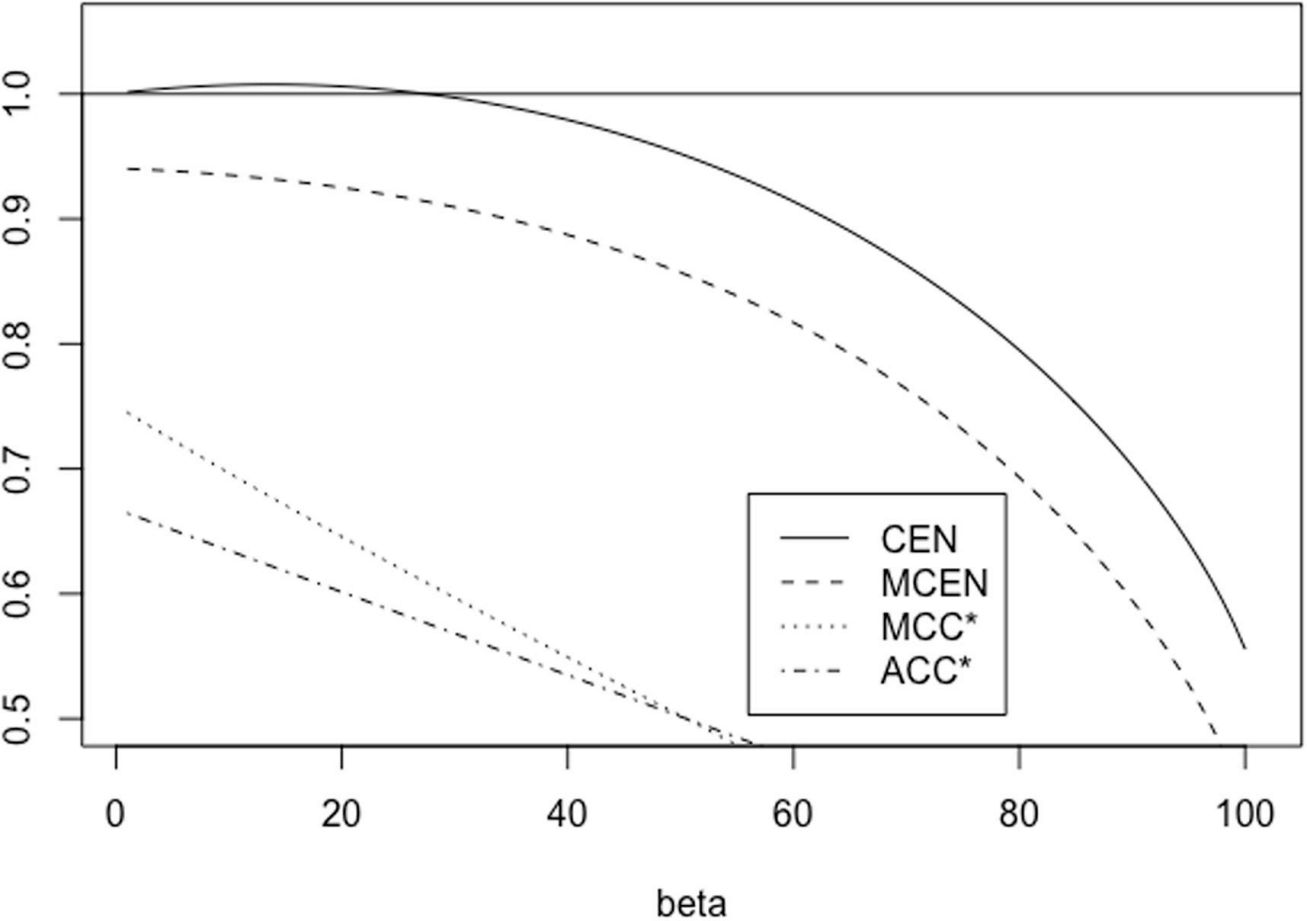
Family $Y_{50,\;2}^{\beta }$ with *β* = 1, 2, …, 101. CEN, MCEN, ACC* and MCC* as function of *β*.

**Example 2:** A similar phenomenon can be observed with family $Y_{100,\;1}^{\beta }=(\begin{array}{cc} 100 & 100\\ 101-\beta & \beta \end{array})$, with *β* = 1, 2, …, 101 (with *β* = 1 the corresponding matrix belongs to the family {*Y*_*A*, *r*_} with *A* = 100 and *r* = 1. As in Example 1, imbalance in classes is constant and when *β* = 1, the minority class is classified very badly, improving classification as *β* increases up to 101, when perfect classification is reached. MCEN as well as ACC* and MCC*, monotonically decrease when *β* increases, while CEN increases up to *β* = 14 and then starts to decrease and behave like the other performance measures (see Fig 15).

### The *Z*_*A*_ family

As noted in [4], the behaviour of the Confusion Entropy CEN is rather diverse from that of MCC* and ACC* for the pathological case of the family of confusion matrices *Z*_*A*_ = (*a*_*i*,*j*_)_*i*,*j* = 1, …, *N*_, defined by $a_{i,j}=\{\begin{array}{cc} A & \;\text{if}\;i=N,\;j=1\\ 1 & \;\text{otherwise,} \end{array}\;$, with *A* > 0. That is, $Z_{A}=(\begin{array}{cccc} 1 & 1 & \ldots & 1\\ 1 & 1 & \ldots & 1\\ \vdots & \vdots & \ldots & \vdots \\ 1 & 1 & \ldots & 1\\ A & 1 & \ldots & 1 \end{array})$. We want to study how MCEN behaves when applied to elements of this family.

**Proposition 7**

$$\begin{array}{cc} If\;N>2,\;\text{CEN}(Z_{A}) & =\frac{1}{N^{2}+A-1}(\left(N-1\right)\;\left(N-2\right)\;\log_{2(N-1)}\left(2N\right)\\ & +\left(2N+A-3\right)\;\log_{2(N-1)}\;\left(2N+A-1\right)-A\;\log_{2(N-1)}\left(A\right)),\\ \text{MCEN} & =\frac{2}{2(N^{2}+A-1)-N}((N-1)(N-2)\log_{2(N-1)}(2N-1)\\ & +(2N+A-3)\log_{2(N-1)}(2N+A-2)-A\log_{2(N-1)}(A)),\\ if\;N=2,\;\text{CEN}(Z_{A}) & =\frac{1}{A+3}\;(\left(A+1\right)\;\log_{2}\left(A+3\right)-A\;\log_{2}\left(A\right)),\\ \text{MCEN} & =\frac{2}{2\;A+5}\;(\left(A+1\right)\;\log_{2}\left(A+2\right)-A\;\log_{2}\left(A\right)). \end{array}$$

*In general (N* ≥ 2),

$$\begin{array}{cc} {\text{MCC}}^{*}\left(Z_{A}\right) & =\frac{N\;(N^{2}+2\;\left(A-1\right))-(N^{2}+\;\left(A-1\right))}{2\;\left(N-1\right)\;(N^{2}+2\;\left(A-1\right))},\\ {\text{ACC}}^{*}\left(Z_{A}\right) & =\frac{N^{2}-N+\left(A-1\right)}{N^{2}+\left(A-1\right)} \end{array}$$

*As a consequence,*

- *If N* = 2,
  MCEN < CEN(Z_A_) *for all* A>0,
  MCEN < 1 *for all* A > 0, *and there exists* A_3_ ∈ (1, 2)(A_3_ ≈ 1.85) *such that*
  $$\begin{array}{cc} & \;\text{CEN}\left(Z_{1}\right)=\text{CEN}\left(Z_{A_{3}}\right)=1,\\ & \;\text{CEN}\left(Z_{A}\right)>1\;if\;A\in \left(1,\;A_{3}\right)\;\;\;and\;\;\;\text{CEN}\left(Z_{A}\right)<1\;\;if\;A\notin \left[1,\;A_{3}\right], \end{array}$$
  $$\begin{array}{cc} & \lim_{A\to 0}{\text{MCC}}^{*}\left(A\right)\;=\;\frac{1}{4}\;<\;\lim_{A\to 0}{\text{ACC}}^{*}\;=\;\frac{1}{3}\;<\;\lim_{A\to 0}\text{MCEN}\left(A\right)\;=\;\frac{2}{5}\;<\;\lim_{A\to 0}\text{CEN}\left(A\right)\;=\;\frac{\log_{2}\left(3\right)}{3},\\ & \lim_{A\to +\infty }\text{CEN}\left(A\right)=\lim_{A\to +\infty }\text{MCEN}\left(A\right)=0<\lim_{A\to +\infty }{\text{MCC}}^{*}=\frac{3}{4}<\lim_{A\to +\infty }{\text{ACC}}^{*}=1\;. \end{array}$$
- *If N* = 3 *(we take this case as example of what happens with N* > 2*),*
  $$\begin{array}{cc} & \lim_{A\to 0}{\text{MCC}}^{*}\left(A\right)=\frac{13}{28}<\lim_{A\to 0}{\text{ACC}}^{*}=\frac{5}{8}<\\ & <\lim_{A\to 0}\text{CEN}\left(A\right)\;=\;\frac{2\;\log_{4}\left(6\right)+3\log_{4}\left(5\right)}{8}\;<\;\lim_{A\to 0}\text{MCEN}\left(A\right)\;=\;\frac{2}{13}\left(2\log_{4}\left(5\right)+3\right)\;<\;1,\\ & \lim_{A\to +\infty }\text{CEN}\left(A\right)\;=\;\lim_{A\to +\infty }\text{MCEN}\left(A\right)\;=\;0\;<\;\lim_{A\to +\infty }{\text{MCC}}^{*}\;=\;\frac{5}{8}\;<\;\lim_{A\to +\infty }{\text{ACC}}^{*}\;=\;1. \end{array}$$

**Fig 16:**
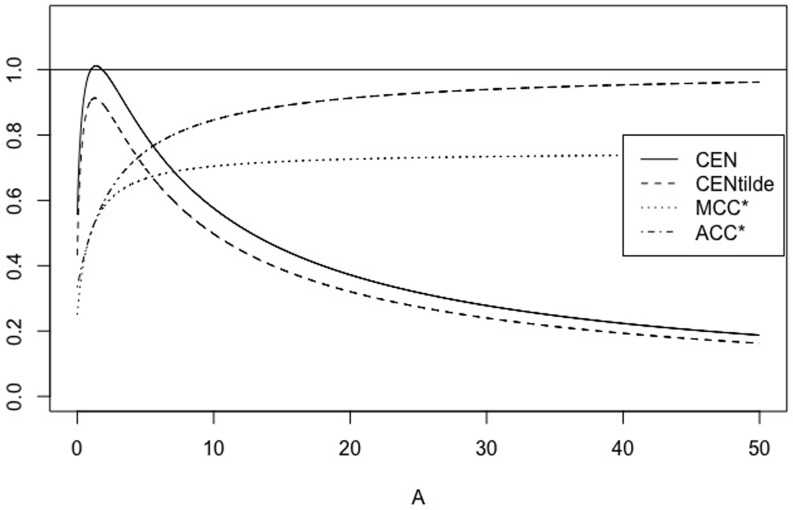
Family *Z*_*A*_. CEN, MCEN, MCC* and ACC* as function of *A* > 0 for *N* = 2.

**Fig 17:**
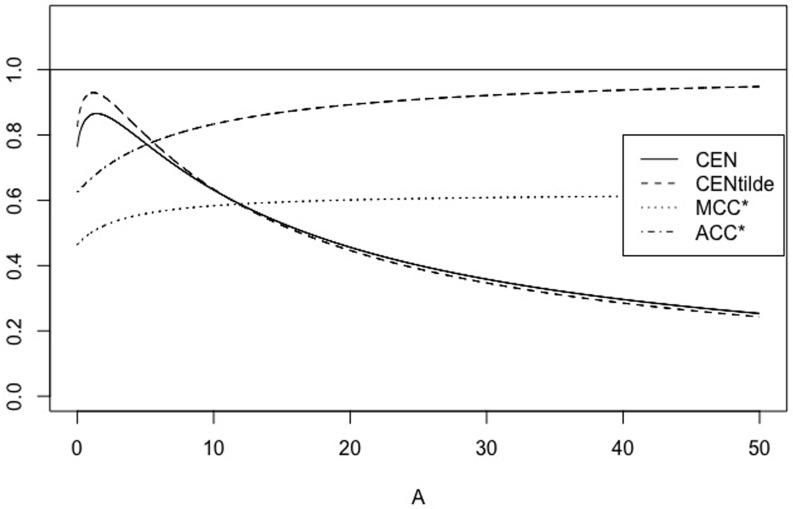
Family *Z*_*A*_. CEN, MCEN, MCC* and ACC* as function of *A* > 0 for *N* = 3.

In Figs 16 and 17 we can observe this behaviour when *N* = 2 and *N* = 3, respectively.

Table 8 shows some examples of confusion matrices of the family *Z*_*A*_, first with *N* = 2, and secondly with *N* = 4.

**Table 8:** Examples with different matrices *Z*_*A*_ in cases *N* = 2 and *N* = 4.

	$\left(\begin{array}{cc} 10 & 10\\ 1 & 10 \end{array}\right)$	$\left(\begin{array}{cc} 2 & 2\\ 1 & 2 \end{array}\right)$	$\left(\begin{array}{cc} 1 & 1\\ 1 & 1 \end{array}\right)$	$\left(\begin{array}{cc} 1 & 1\\ 2 & 1 \end{array}\right)$	$\left(\begin{array}{cc} 1 & 1\\ 10 & 1 \end{array}\right)$
A =	1/10	1/2	1	2	10
ACC* =	0.3548	0.4286	0.5000	0.6000	0.8462
MCC* =	0.2955	0.4167	0.5000	0.5833	0.7045
CEN =	0.6864	0.9174	1.0000	0.9932	0.5758
MCEN =	0.5806	0.8276	0.9057	0.8889	0.4972
*Z*_*A*_ =	$\left(\begin{array}{cccc} 10^{2} & 10^{2} & 10^{2} & 10^{2}\\ 10^{2} & 10^{2} & 10^{2} & 10^{2}\\ 10^{2} & 10^{2} & 10^{2} & 10^{2}\\ 1 & 10^{2} & 10^{2} & 10^{2} \end{array}\right)$	$\left(\begin{array}{cccc} 10 & 10 & 10 & 10\\ 10 & 10 & 10 & 10\\ 10 & 10 & 10 & 10\\ 1 & 10 & 10 & 10 \end{array}\right)$	$\left(\begin{array}{cccc} 1 & 1 & 1 & 1\\ 1 & 1 & 1 & 1\\ 1 & 1 & 1 & 1\\ 1 & 1 & 1 & 1 \end{array}\right)$	$\left(\begin{array}{cccc} 1 & 1 & 1 & 1\\ 1 & 1 & 1 & 1\\ 1 & 1 & 1 & 1\\ 10 & 1 & 1 & 1 \end{array}\right)$	$\left(\begin{array}{cccc} 1 & 1 & 1 & 1\\ 1 & 1 & 1 & 1\\ 1 & 1 & 1 & 1\\ 10^{2} & 1 & 1 & 1 \end{array}\right)$
A =	10^−2^	10^−1^	1	10	10^2^
ACC* =	0.7335	0.7351	0.7500	0.8400	0.9652
MCC* =	0.4882	0.4894	0.5000	0.5441	0.5771
CEN =	0.8284	0.8391	0.8704	0.7132	0.2068
MCEN =	0.8883	0.9001	0.9309	0.7338	0.2016

Note that CEN and MCEN exhibit a very different behaviour comparing with ACC* and MCC*, since the former are sensitive to the overall entropy associated to the elements of the matrix, which is $\log\left(N^{2}+A-1\right)-\frac{A}{N^{2}+A-1}\;\log\left(A\right)$. Entropy decreases to log(*N*^2^ − 1) when *A* → 0, and drops to 0 when *A* → +∞.

## Comparing with other performance measures

Several works have considered the question of the introduction and comparison of different performance measures for classification, inspired, in one way or another, by Shannon’s entropy. For example, in [13] the authors introduce a novel measure called PACC (Probabilistic Accuracy) in the multi-class setting, making a comparative study of it with other measures as Accuracy, MCC and CEN, among others.

Besides, Entropy-Modulated Accuracy (EMA), introduced in [14], is a performance measure of classification tasks based on the concept of *perplexity*, the latter being defined as the effective number of classes a classifier sees. The authors also introduce NIT (Normalized Information Transfer) factor, which is a correction of EMA. They compare both EMA and NIT factor with Accuracy and CEN, rejecting rankings of classifiers based in Accuracy and choosing more meaningful and interpretable classifiers. They show in some examples that MCC is highly correlated with Accuracy, while rankings obtained with CEN, EMA and NIT factor are comparable in some cases but disagree in others.

Although PACC, EMA and NIT factor are useful measures to assess classifiers, in our opinion none of them is completely satisfactory in grading the effectiveness of the classifier learning process, since all reflect some concrete feature of the classification process, being insufficient for covering all the aspects of this complex task, so they should be used cautiously and in a complementary way. That is, all the measures suffer from certain weaknesses that are evident in specific, more or less gimmicky situations. This comment extends also to both CEN and MCEN, although it should be noted that the latter solves the problems showed by CEN in the binary setting, as well as to MCC and Accuracy, the last one having been widely treated (see, for example, the Introduction section in [14]).

Let us exemplify this fact by going back to the toy example in Table 2. In Table 9 we add the calculated values of PACC* = 1-PACC and 1/NIT to that of Table 2. We use NIT factor (inverted to make it comparable with the other measures) instead of EMA since the probability distribution of classes in the validation set is not uniform. Note that our confusion matrices are transposed with respect to that in [14], and also that for the NIT factor we use formula (4). We have used the corrected definition provided by the authors, which had already acknowledged an erratum in Eq (4) in the comments of https://www.researchgate.net/publication/259743406_100_Classification_Accuracy_Considered_Harmful_The_Normalized_Information_Transfer_Factor_Explains_the_Accuracy_Paradox/.

**Table 9:** Toy example of Table 2 revisited, adding PACC and the NIT factor.

	Baseline	(a)			(b)		
	$\left(\begin{array}{cc} 3 & 3\\ 3 & 3 \end{array}\right)$	$\left(\begin{array}{cc} 2 & 3\\ 3 & 4 \end{array}\right)$	$\left(\begin{array}{cc} 1 & 3\\ 3 & 5 \end{array}\right)$	$\left(\begin{array}{cc} 0 & 3\\ 3 & 6 \end{array}\right)$	$\left(\begin{array}{cc} 3 & 2\\ 4 & 3 \end{array}\right)$	$\left(\begin{array}{cc} 3 & 1\\ 5 & 3 \end{array}\right)$	$\left(\begin{array}{cc} 3 & 0\\ 6 & 3 \end{array}\right)$
ACC* =	0.5000	0.5000	0.5000	0.5000	0.5000	0.5000	0.5000
MCC* =	0.5000	0.5130	0.5625	0.6667	0.4881	0.4375	0.3333
CEN =	1.0000	0.9898	0.9575	0.8962	0.9591	0.8250	0.5000
MCEN =	0.9057	0.9006	0.8848	0.8571	0.8590	0.7057	0.3343
PACC* =	0.5000	0.5071	0.5312	0.5833	0.4929	0.4687	0.4167
1/NIT =	2.0000	1.9992	1.9840	1.8371	1.9992	1.9840	1.8371

The behaviour of PACC* showed in Table 9 is consistent with that of MCC*, increasing when IN entropy decreases (a) and decreasing when OUT decreases (b). However, the behaviour of 1/NIT is consistent with that of CEN and MCEN, decreasing in both cases. Nevertheless, unlike what happens with CEN and MCEN, NIT factor does not distinguish among scenarios (a) and (b). This is because both EMA and NIT factor are invariants to permutations of the columns.

Another example is that of the *MEG mind reading challenge* organized by the PASCAL (Pattern Analysis, Statistical modeling and ComputAtional Learning) network in [15], already considered in [14]. We restrict our comparison to the group of the four most outstanding systems, denoted *C*_1_ (Huttunen et al.), *C*_2_ (Santana et al.), *C*_3_ (Jylänki et al.) and *C*_4_ (Tu & Sun), since for them, unlike what happens with the rest, we could access to the confusion matrices in [15]. The results are in Table 10, and from them we see that the most comparable rankings are that given by the NIT factor, CEN and MCEN, showing clusters {*C*_4_, *C*_2_} and {*C*_1_, *C*_3_}, with very small differences inside the clusters, specially the second. The authors of the report [15] were specially interested in comparison *C*_1_ vs. *C*_4_, and 1/NIT factor, as well as CEN and MCEN, give the same ordering: *C*_4_ is better (lower value) than *C*_1_, in concordance with interpretability given in [14].

**Table 10:** Results for the first four systems of the *MEG mind reading challenge*. Confusion matrices have been obtained from [15].

System	ACC*	MCC*	CEN	MCEN	PACC*	1/NIT
*C*_1_	0.3201	0.2010	0.4360	0.5694	0.3230	2.5877
*C*_2_	0.3675	0.2286	0.4043	0.4981	0.3668	2.4715
*C*_3_	0.3721	0.2319	0.4483	0.5645	0.3667	2.6151
*C*_4_	0.3783	0.2369	0.4213	0.5279	0.3737	2.4545

One more example to show the variability when performance measures are compared: in Table 11 we see that the NIT factor (equivalently, EMA), unlike the other measures, is not able to distinguish between classifiers whose confusion matrices are *A* and *B* in the binary case, nor between *C* and *D* in multi-class classification.

**Table 11:** Two toy examples. With *S* = 30 for *N* = 2, and with *S* = 40 for *N* = 3.

	$A=(\begin{array}{cc} 10 & 0\\ 10 & 10 \end{array}\;)$		$B=\left(\begin{array}{cc} 0 & 10\\ 10 & 10 \end{array}\;\right)$	$C=(\begin{array}{ccc} 10 & 0 & 0\\ 10 & 10 & 0\\ 0 & 0 & 10 \end{array}\;)$		$D=\left(\begin{array}{ccc} 10 & 0 & 0\\ 0 & 10 & 10\\ 10 & 0 & 0 \end{array}\;\right)$
ACC* =	0.3333	<	0.6667	0.2500	<	0.5000
MCC* =	0.2500	<	0.7500	0.1500	<	0.3500
CEN =	0.5283	<	1.0000	0.1981	<	0.3231
MCEN =	0.4000	<	0.9400	0.2000	<	0.3333
PACC* =	0.2917	<	0.7083	0.1944	<	0.5000
1/NIT =	1.6799	=	1.6799	1.5000	=	1.5000

## Supporting information file: Experiments and results

The advantages of using Modified Confusion Entropy MCEN measure against CEN have been tested on different binary classifiers, constructed from four available datasets from the UCI ML Repository (https://archive.ics.uci.edu). From each dataset we construct and assess eight different classifiers, five of which are Bayesian networks, while the rest are other standard machine learning procedures used in supervised classification problems.

Because of the comparisons carried out previously with different examples, we have to recognize the impossibility of deciding what measure of behaviour, of the considered ones, can allow to decide in the case that the rankings of classifiers obtained with CEN and MCEN were different. We decided, then, to use OUT entropy as such a reference when there is disparity; in case of a tie, we will use IN entropy to break it. This is what we will call “the criterion of entropy”.

To compare rankings obtained from CEN and MCEN and that obtained by the criterion of entropy, we use both the Hamming distance and the degree of consistency indicator *c* (see [16]).

The results obtained with all the considered datasets heuristically reinforce that MCEN is more correlated with entropy than CEN. (see S1 File and Tables A-F in S1 File).

## Conclusion

We introduced MCEN as a modification of the original Confusion Entropy performance measure CEN introduced in [3], both for binary and multi-class classification, proving some properties. We compared this measure with CEN, MCC and Accuracy, showing that in the binary case, MCEN overcomes the unreliability of CEN in a twofold sense: the departure of the range where it should be (the interval [0, 1]), and the lack of monotonicity when the entropy increases or decreases. These features made CEN an inappropriate measure in the binary case, proving MCEN to be a good alternative, and we study different scenarios to highlight this fact. Moreover, while nor Accuracy nor MCC can distinguish among different misclassification distributions of cases in the confusion matrix, MCEN and CEN have an high level of discrimination.

First, we show that in the binary case (see Table 2), both CEN and MCEN are sensitive to the decreasing in the entropy within the main diagonal IN, an also to that outside the diagonal OUT, but while CEN is more sensitive than MCEN to IN, the opposite occurs with OUT. By contrast, ACC is insensitive as long as the sum of the diagonal and the total sum remain constant. Secondly, we consider the multi-class perfectly symmetric and balanced case in which the main diagonal elements are equal to *T* and the elements outside the diagonal are equal to *F*, which is analytically studied in detail, showing the output-of-range of CEN in the binary case when *γ* = *T*/*F* ∈ (0, 1).

After that, se consider different particular situations in the binary setting, through the study of some families of confusion matrices. Family *U*_*A*_ is symmetric and unbalanced, showing the out-of-range of CEN for any *A* > 1, and in addition a lack of monotonicity that contrast with the behaviour of the overall entropy associated to the elements of the matrix. Family *V*_*A*_ is asymmetric and unbalanced, and also shows the out-of-range of CEN but only for *A* in the interval (1, *A*_1_), where *A*_1_ ≈ 1.4.

Two doubly indexed families have been considered in the binary case. CEN has an anomalous behaviour for family *X*_*A*, *r*_, which is symmetric but unbalanced, for *r* > *r*_0_ (with *r*_0_ ≈ 0.8) since it is not only out-of-range from a certain value of *A*, but its limit when *A* → +∞ is >1 if *r* > 1, showing lack of monotonicity. The same happens from a certain value of *r*, fixed *A*. Family *Y*_*A*, *r*_ is also unbalanced but asymmetric. When *r* is in the interval (*R*_0_, 1) with *R*_0_ ≈ 0.71, CEN is not only out-of-range from a certain value of *A*, but its limit when *A* → +∞ is >1 if *r* > 1, showing lack of monotonicity. But there are other two intervals of values for *r* in which CEN>1 for *A* living in a certain bounded interval.

Besides evaluating binary confusion matrices with the same classification results for the minority class but different balances of the two classes, we compare through two examples the behaviour of MCEN with that of CEN, ACC* and MCC*, in evaluating improvements in classification of the minority class while maintaining the same amount of imbalance. We show that CEN is the only one that does not show a monotonous decrease as the classification improves, for which MCEN proves, also in this sense, that it outperforms CEN.

Finally, we also consider the multi-class family *Z*_*A*_, which is asymmetric and unbalanced, and observe that in the binary case, CEN is out-of-range for *A* ∈ (1, *A*_3_), with *A*_3_ ≈ 1.85.

In all of these examples, MCEN behave appropriately. Comparing with the overall Shannon’s entropy associated to the set of elements of the confusion matrix, both CEN and MCEN are sensitive to it but CEN sometimes does not show the same behaviour in terms of monotonicity than entropy. With respect to Accuracy and MCC, conveniently scaled, they show sometimes a behaviour in contradiction with Shannon’s entropy, as for families *V*_*A*_ and *Z*_*A*_.

A further comparison has been carried out with the Probabilistic Accuracy (PACC) introduced in [13], and the Entropy-Modulated-Accuracy EMA and the Normalized Information Transfer (NIT) factor, both introduced in [15]. We consider different examples in which sometimes PACC* = 1–PACC behaves consistently with MCC*, increasing when IN entropy decreases and decreasing when OUT decreases, while 1/NIT behaves in accordance with CEN and MCEN, decreasing in both cases, but with the handicap that unlike what happens with CEN and MCEN, NIT factor does not distinguish between IN and OUT. But not always. Actually, no measure seems to be completely satisfactory since each one reflects a specific characteristic of the classification process, so they should be used in a complementary way and none can be taken as a gold standard to compare the others.

Finally, to make clear the improvement of MCEN over CEN, we carry out experimentation consisting in the comparison of the rankings of some classifiers obtained from four different real datasets by using both measures. Mostly the classifiers orderings match, but when they do not, it is the MCEN that most agrees with the criterion of entropy. To see that, we use both the Hamming distance and the degree of consistency indicator c. These results heuristically support the use of MCEN as a better alternative to CEN in the binary case, when a performance measure based in entropy is required.

## Supporting information

S1 File Supporting information: Experiments and results. Table A. Datasets used in the experiments. Table B. Classifiers used in the experiments. Table C. Results for the Breast cancer dataset. Table D. Results for the SPECT heart dataset. Table E. Results for the Congressional voting dataset. Table F in S1 File. Results for the MONK’s Problems.
(PDF)

S2 File Breast cancer dataset. (CSV)

S3 File Breast cancer description. (PDF)

S4 File SPECT dataset. (CSV)

S5 File SPECT description. (PDF)

S6 File UCB admissions dataset. (CSV)

S7 File UCB admissions description. (PDF)
