# Correction: Enhancing Confusion Entropy (CEN) for binary and multiclass classification

**DOI:** 10.1371/journal.pone.0250834

**Published:** 2021-04-22

**Authors:** Rosario Delgado, J. David Núñez-González

One of the affiliations for the second author is not indicated. J. David Núñez-González is affiliated with: Autonomous University of Barcelona (UAB).

## References

[1] Delgado R, Núñez-González JD (2019) Enhancing Confusion Entropy (CEN) for binary and multiclass classification. PLoS ONE 14(1): e0210264. 10.1371/journal.pone.0210264 30640948 PMC6331113

