# Supplementary material for: Enhancing Confusion Entropy (CEN) for binary and multiclass classification
Source: PLoS One. 2019 Jan 14;14(1):e0210264. doi: 10.1371/journal.pone.0210264 (PMC6331113; doi:10.1371/journal.pone.0210264)
Supplement: S1 File — Table A. Datasets used in the experiments. Table B. Classifiers used in the experiments. Table C. Results for the Breast cancer dataset. Table D. Results for the SPECT heart dataset. Table E. Results for the Congressional voting dataset. Table F in S1 File. Results for the MONK’s Problems. (PDF) [file pone.0210264.s001.pdf]

Enhancing Confusion Entropy (CEN) for binary and multiclass classification

Rosario Delgado[1], J. David Núñez-González[2*],

1 Department of Mathematics
Universitat Autònoma de Barcelona
Campus de la UAB, 08193 - Cerdanyola del Vallès, Spain
2 Department of Mathematics
University of the Basque Country (UPV/EHU), Leioa, Spain

* E-mail: josedavid.nunez@ehu.eus. Corresponding author.

Supporting Information: Experiments and Results

The advantages of using Modified Confusion Entropy MCEN measure against CEN have been tested on different binary classifiers, constructed from some available datasets, each of them with different number of categorical features. The class variable of any of the datasets is binary, which is the case when dysfunctionality of CEN can be highlighted and MCEN can show its comparatively better behaviour. However, it should be noted that in most cases both measures behave very similarly. The datasets we use are all public domain, from the UCI ML Repository (https://archive.ics.uci.edu), so that the results can be reproduced. Information on the original datasets can be found in Table A.

Dataset	# cases	# features	Classes	Ambit
Breast cancer	286	9	no-recurrence events/recurrence events	Oncology
SPECT heart	267	22	normal/abnormal	Diagnosis by the image
Congressional Voting	435	16	democrat/republican	Social Sciences
MONK's Problems	432	6	robot belongs to the class/does not belong	Artificial Intelligence

Table A. Datasets used in the experiments.

From each dataset we construct and assess the classifiers shown in Table B, the first five being Bayesian networks, while the rest are other standard machine learning procedures used in supervised classification problems.

Classifier	Notation
Naive Bayes	BC1
Bayesian network. BIC score. Restriction of no directed edges from features to the class	BC2
Bayesian network. AIC score. Restriction of no directed edges from features to the class	BC3
Augmented Naive Bayes from BC1. AIC score. Allows directed edges among features	BC4
Tree Augmented Naive (TAN)	BC5
Neural network	NN
Support vector machine	SVM
Random forest	RF

Table B. Classifiers used in the experiments.

The Bayesian Information Criterion (BIC) score is used to construct classifier BC2, and it is defined as $BIC(\Gamma,\, D) = \ln\left(L^D(\theta^{\Gamma}_{MLE})\right) - \frac{d}{2}\ln(M)$, where Γ denotes the Directed Acyclic Graph (DAG) associated to the Bayesian network, D is the training dataset used for learning the model, composed of M cases, $L^D(\theta^{\Gamma}_{MLE})$ denotes the estimated Likelihood Function associated to the model when the Maximum Likelihood Estimation of the parameters is used, and d denotes the measure of complexity of the model, that is, the number of non-redundant parameters. The Akaike score (AIC), which is used for construction of classifier BC3, is defined as $AIC(\Gamma,\, D) = \ln\left(L^D(\theta^{\Gamma}_{MLE})\right) - d$. AIC is a score that penalizes for complexity less than BIC if M is big enough (indeed, if $M > 7$). Therefore, the use of AIC as score function gives rise to DAGs more connected than that obtained using the BIC score.

We use language R [1] and different packages of R: "bnlearn" [2] and"gRain" [3] for the first five classifiers, and "mlearning" [4] for the rest.

As a result of the comparisons carried out previously with different examples, we have to recognize the impossibility of deciding what measure of behaviour, of the considered ones, can allow to decide in the case that the rankings of classifiers obtained with CEN and MCEN were different. We decided, then, to use OUT entropy as such a reference when there is disparity; in case of a tie, we will use IN entropy to break it. This is what we will call in what follows "the criterion of entropy".

To compare rankings obtained from CEN and MCEN and that obtained by the criterion of entropy, we use both the Hamming distance and the degree of consistency indicator c (see [5]). In information theory, the Hamming distance between two strings of equal length is the number of positions at which the corresponding symbols are different, that is, it measures the minimum number of substitutions required to change one string into the other. On the other hand, for two measures f and g on a domain Ψ, let us define

$$R = \{(a,\, b) \in \Psi \times \Psi \,:\, f(a) > f(b),\, g(a) > g(b)\}$$
$$V = \{(a,\, b) \in \Psi \times \Psi \,:\, f(a) > f(b),\, g(a) < g(b)\}\,.$$

Then, the *degree of consistency c of f and g* is defined by $c(f,\, g) = \frac{|R|}{|R|+|V|}$, where $|A|$ denotes the number of elements of the (finite) set A. We apply this indicator with f and g ranking functions associated to CEN (respect. MCEN) and the entropy criterion.

Breast cancer dataset

This dataset has been provided by the Oncology Institute of the University Medical Center of Ljubljana, Slovenia, and is available from the UCI Machine Learning repository [6], containing 286 cases, 201 of one class ("no-recurrence-events") and 85 of the other ("recurrence-events"). The cases are described by 9 features, some of them nominal, and the rest discrete, which include age, tumor-size, breast (left/(right) and irradiated (yes/no). We performed $k-$fold cross-validation with $k = 10$, taking as seed for division into folders the first random value between 1 and 1000 generated from the seed 666 by R, that is, 775. Then, we learn the eight classifiers in Table B, and validate them, obtaining the corresponding confusion matrices. We rank the classifiers for each of the 10 folders, by using CEN and MCEN. To compare these two measures, we also rank the classifiers using the criterion of entropy. We obtain results in Table C.

Folder	Measure	Rankings	Hamming distance	Consistency indicator c
1	CEN	BC4>RF>BC1>BC3>BC5>NN>**BC2**>**SVM**	5	22/27
✓	MCEN	BC4>RF>BC1>BC3>BC5>NN>**SVM**>**BC2**	4	23/27
	Entropy	RF>BC4>BC5>BC1>BC3>**SVM**>**BC2**=NN		
2	CEN	RF>BC4>BC1>BC5>BC3>SVM>BC2>NN	2	25/27
=	MCEN	RF>BC4>BC1>BC5>BC3>SVM>BC2>NN	2	25/27
	Entropy	RF>BC1>BC4>BC5>SVM>BC3>BC2=NN		
3	CEN	BC5>BC1>BC4>RF>BC3>SVM>BC2>NN	2	25/27
=	MCEN	BC5>BC1>BC4>RF>BC3>SVM>BC2>NN	2	25/27
	Entropy	BC5>RF>BC1>BC4>BC3>SVM>BC2=NN		
4	CEN	**BC5**>**BC3**>BC4=RF>BC1>SVM>BC2=NN	3	20/26
×	MCEN	**BC3**>**BC5**>BC4=RF>BC1>SVM>BC2=NN	4	19/26
	Entropy	**BC5**>BC4=RF>BC1>**BC3**>SVM>BC2=NN		
5	CEN	BC1>BC3>**BC5**>**BC4**>RF>BC2>NN=SVM	3	22/26
✓	MCEN	BC1>BC3>**BC4**>**BC5**>RF>BC2>NN=SVM	2	23/26
	Entropy	BC1>**BC4**>BC3>RF>**BC5**>BC2=NN=SVM		
6	CEN	BC5>BC3>BC4>BC1>**NN**>**SVM**=**RF**>BC2	2	23/25
✓	MCEN	BC5>BC3>BC4>BC1>**SVM**=**RF**>**NN**>BC2	0	25/25
	Entropy	BC5>BC3=BC4>BC1>**SVM**=**RF**>BC2=**NN**		
7	CEN	NN>BC1=BC3=BC4>BC2>**RF**>**BC5**>SVM	17	9/25
×	MCEN	NN>BC1=BC3=BC4>BC2>**BC5**>**RF**>SVM	18	8/25
	Entropy	**RF**>BC2>**BC5**>BC1=BC3=BC4>NN>SVM		
8	CEN	BC5>BC1>BC4>RF>**SVM**>**NN**>**BC3**>BC2	6	19/27
✓	MCEN	BC5>BC1>BC4>RF>**BC3**>**SVM**>**NN**>BC2	4	20/27
	Entropy	**BC3**>BC5>BC1>BC4>RF>**SVM**>BC2=**NN**		
9	CEN	**BC5**>**BC4**>BC3>BC1>SVM>RF>BC2=NN	1	26/27
✓	MCEN	**BC4**>**BC5**>BC3>BC1>SVM>RF>BC2=NN	0	27/27
	Entropy	**BC4**>**BC5**>BC3>BC1>SVM>RF>BC2=NN		
10	CEN	BC5>BC4>BC1>RF>SVM>NN>BC3>BC2	1	26/27
=	MCEN	BC5>BC4>BC1>RF>SVM>NN>BC3>BC2	1	26/27
	Entropy	BC5=BC1>BC4>RF>SVM>NN>BC3>BC2		

Table C. Results for the Breast cancer dataset. In boldface classifiers ranked differently with CEN and MCEN.

We observe that in cases where rankings given by CEN and MCEN are different (7 out of 10), Hamming distance is minimized by MCEN in 5 out of 7, and the degree of consistency is greater for MCEN in the same cases. This reinforces the hypothesis that MCEN is more correlated with entropy generated by misclassification than CEN.

SPECT heart dataset

The dataset describes diagnosing of cardiac Single Proton Emission Computed Tomography (SPECT) images of 267 patients, that were classified into two categories: normal and abnormal. From the original SPECT images, and after an extraction process, 22 binary features were consigned. The original owners of the dataset are from the Medical College of Ohio, OH, U.S.A., and it is available from the UCI Machine Learning repository [6]. We performed $k-$fold cross-validation with $k = 10$, using the same seed as in the previous example, that is, 775, and we learn classifiers in Table B, and validate them. The rankings of these classifiers by CEN, MCEN and the criterion of entropy are in Table D. As before, to compare rankings we use both the Hamming distance and the degree of consistency indicator c.

Folder	Measure	Rankings	Hamming distance	Consistency indicator c
1	CEN	BC3>BC4>**BC5**>**RF**>BC1>BC2=NN=SVM	3	22/25
×	MCEN	BC3>BC4>**RF**>**BC5**>BC1>BC2=NN=SVM	4	21/25
	Entropy	BC4>**BC5**>**RF**>BC3>BC1>BC2=NN=SVM		
2	CEN	BC5>BC3>BC4>RF>SVM>BC1>BC2>NN	4	22/26
=	MCEN	BC5>BC3>BC4>RF>SVM>BC1>BC2>NN	4	22/26
	Entropy	BC5>BC1>BC3>BC4=RF>SVM>BC2=NN		
3	CEN	BC2>BC3>BC5>RF>BC4>SVM>NN>BC1	12	16/28
=	MCEN	BC2>BC3>BC5>RF>BC4>SVM>NN>BC1	12	16/28
	Entropy	SVM>BC3>BC4>RF>BC2>BC5>BC1>NN		
4	CEN	BC5>BC1>RF>**NN**>**BC3**>BC4>BC2>SVM	4	24/28
✓	MCEN	BC5>BC1>RF>**BC3**>**NN**>BC4>BC2>SVM	3	25/28
	Entropy	BC1>BC5>RF>**BC3**>BC4 >**NN**>SVM>BC2		
5	CEN	BC1>BC5=SVM>BC4>BC2=NN=RF>BC3	0	17/17
=	MCEN	BC1>BC5=SVM>BC4>BC2=NN=RF>BC3	0	17/17
	Entropy	BC1>BC5=SVM>BC4=BC2=NN=RF=BC3		
6	CEN	BC2>**BC4**>**BC1**>**BC3**>**NN**=**RF**>SVM>BC5	17	12/27
✓	MCEN	BC2>**BC3**>**BC4**>**NN**=**RF**>**BC1**>SVM>BC5	11	16/27
	Entropy	**BC3**>SVM>**NN**=**RF**>**BC4**>BC2>**BC1**>BC5		
7	CEN	**BC1**>**BC3**>**BC2**=**NN**>**BC4**=**BC5** >SVM>RF	8	4/12
✓	MCEN	**BC4**=**BC5**>**BC1**>**BC3**>**BC2**=**NN**>SVM>RF	0	12/12
	Entropy	**BC4**=**BC5**>**BC1**=**BC3**=**BC2**=**NN**=SVM=RF		
8	CEN	BC4>BC1>SVM=RF>**BC2**>**NN**>**BC5**>**BC3**	8	17/25
✓	MCEN	BC4>BC1>SVM=RF>**BC5**>**BC3**>**BC2**>**NN**	4	21/25
	Entropy	**BC5**>BC4=BC1>SVM=RF>**BC3**>**BC2**=**NN**		
9	CEN	NN>BC3>BC5>BC4>RF>BC1>SVM>BC2	3	24/26
=	MCEN	NN>BC3>BC5>BC4>RF>BC1>SVM>BC2	3	24/26
	Entropy	BC3> NN>BC4>BC5=RF>SVM>BC1>BC2		
10	CEN	BC4>BC3>RF>**BC5**>**SVM**>BC1>BC2>NN	6	7/13
✓	MCEN	BC4>BC3>RF>**SVM**>**BC5**>BC1>BC2>NN	5	8/13
	Entropy	**SVM**>RF>BC4=BC3=**BC5**=BC1=BC2=NN		

Table D. Results for the SPECT heart dataset. In boldface classifiers ranked differently with CEN and MCEN.

We observe from Table D that when rankings given by CEN and MCEN are different (6 of 10 cases), Hamming distance is minimized by MCEN in 5 out of 6 cases, being the degree of consistency greater for MCEN in the same cases. Heuristically we get the same conclusion as with the Breast cancer dataset.

Congressional voting dataset

This dataset includes votes for each of the 435 members of the U.S. House of Representatives on the 16 key votes identified by the CQA (Congressional Quarterly Almanac), which is also the source (CQA, 98th Congress, 2nd session 1984, Volume XL: Congressional Quarterly Inc. Washington, D.C., 1985). All attributes are binary, with values "yes" (meaning voted for, paired for or announced for), "no" (simplification of voted against, paired against or announced against), and missing (otherwise). The predicted class from attributes is the party affiliation. We performed $k-$fold cross-validation with $k = 10$, using the same seed as in the previous datasets (775), and we learn classifiers in Table B, and validate them. The rankings of these classifiers by CEN, MCEN and the criterion of entropy are in Table E, and again we use both the Hamming distance and the degree of consistency indicator c.

Folder	Measure	Rankings	Hamming distance	Consistency indicator c
1	CEN	BC1>BC2>SVM=RF>BC3>BC4>BC5>NN	5	20/27
=	MCEN	BC1>BC2>SVM=RF>BC3>BC4>BC5>NN	5	20/27
	Entropy	BC2>BC3>SVM=RF>BC1>BC5>BC4>NN		
2	CEN	BC5>BC2>BC4>BC1>**NN**>**SVM**=**RF**>BC3	6	14/27
✓	MCEN	BC5>BC2>BC4>BC1>**SVM**=**RF**>**NN**>BC3	5	16/27
	Entropy	BC2>**SVM**=**RF**>BC1>BC4>BC5>BC3>**NN**		
3	CEN	BC1>BC2>SVM=RF>BC3>BC4=BC5>NN	6	18/26
=	MCEN	BC1>BC2>SVM=RF>BC3>BC4=BC5>NN	6	18/26
	Entropy	SVM=RF>BC2>BC1>NN>BC3>BC4=BC5		
4	CEN	BC1>NN>RF>BC3>BC4=BC5>BC2>SVM	15	23/27
=	MCEN	BC1>NN>RF>BC3>BC4=BC5>BC2>SVM	15	23/27
	Entropy	RF>BC1>NN>BC4=BC5>BC3>BC2 >SVM		
5	CEN	BC1>NN>BC3>BC4>BC2>BC5>SVM=RF	7	20/27
=	MCEN	BC1>NN>BC3>BC4>BC2>BC5>SVM=RF	7	20/27
	Entropy	BC1>BC4>BC3>BC5>NN>SVM=RF>BC2		
6	CEN	**BC1**>**BC2**>BC4>BC5>BC3>NN>SVM=RF	4	22/26
✓	MCEN	**BC2**>**BC1**>BC4>BC5>BC3>NN>SVM=RF	3	23/26
	Entropy	**BC2**>BC4>BC3>**BC1**=BC5>NN>SVM=RF		
7	CEN	BC1>NN>BC3>BC4>BC5>BC2>SVM=RF	10	17/27
=	MCEN	BC1>NN>BC3>BC4>BC5>BC2>SVM=RF	10	17/27
	Entropy	BC1>BC3>BC4>SVM=RF>BC5>BC2>NN		
8	CEN	BC4>BC5>NN>BC1>BC2>BC3>SVM=RF	20	7/27
=	MCEN	BC4>BC5>NN>BC1>BC2>BC3>SVM=RF	20	7/27
	Entropy	SVM=RF>NN>BC3>BC5>BC4>BC2>BC1		
9	CEN	BC1>NN>BC4>BC5>BC3>BC2=SVM=RF	4	21/25
=	MCEN	BC1>NN>BC4>BC5>BC3>BC2=SVM=RF	4	21/25
	Entropy	BC4>BC5>BC1>NN>BC3>BC2=SVM=RF		
10	CEN	BC1>BC5>BC4>BC2=BC3=NN=SVM>RF	2	20/22
=	MCEN	BC1>BC5>BC4>BC2=BC3=NN=SVM>RF	2	20/22
	Entropy	BC4>BC1>BC5>BC2=BC3=NN=SVM>RF		

Table E. Results for the Congressional voting dataset. In boldface classifiers ranked differently with CEN and MCEN.

CEN and MCEN give different rankings among the classifiers only in 2 of the 10 cases (see Table E), but in both MCEN minimizes Hamming distance and maximizes the degree of consistency, leading us to the same conclusion as with the previous datasets.

The MONK's Problems

The MONK's problems were the basis of a first international comparison of learning algorithms, and they rely on the artificial robot domain, in which robots are described by six different attributes:

Head shape: round/square/octagon

Body shape: round/square/octagon

Smailing: yes/no

Holding: sword/balloon/flag

Jacket color: red/yellow/green/blue

Tie: yes/no

The learning task of the robots is a binary classification task. Three different problems are considered in this situation, and each one is described by a logical description of the class, and robots belong either to this class or not. Only a subset of all 432 possible robots with its classification is given.

- **Problem 1. Logical description of the class: (Head shape = Body shape) or (Jacket color = red)**

  From 432 possible robots, 124 were randomly selected for the training set, and there were no misclassifications.

- **Problem 2. Logical description of the class: (Exactly two of the six attributes have their first value)**

  From 432 possible robots, 169 were randomly selected for the training set, and again there were no misclassifications.

- **Problem 3. Logical description of the class: (Jacket color=green) or (Jacket color ≠ blue and Body shape ≠ octagon)**

  From 432 possible robots, 122 were randomly selected for the training set, and among them there were 5% misclassifications. This problem serves to evaluate the classifiers under the presence of noise in the training set.

After learning, we validate the classifiers using as validation set the dataset of the 432 robots for each problem separately. The rankings of the classifiers by using CEN, MCEN and the criterion of entropy are in Table F.

Problem	Measure	Rankings	Hamming distance	Consistency indicator c
1	CEN	BC1>SVM>NN>RF>BC5>BC4>BC2=BC3	8	19/27
=	MCEN	BC1>SVM>NN>RF>BC5>BC4>BC2=BC3	8	19/27
	Entropy	SVM>RF>BC1>NN>BC2=BC3>BC4>BC5		
2	CEN	BC4>BC5>BC1>SVM>RF>NN>BC2=BC3	5	22/27
=	MCEN	BC4>BC5>BC1>SVM>RF>NN>BC2=BC3	5	22/27
	Entropy	RF>BC4>BC5>SVM>BC1>NN>BC2=BC3		
3	CEN	NN>**SVM**>**BC5**>RF>BC1=BC2=BC3>BC4	2	23/25
✓	MCEN	NN>**BC5**>**SVM**>RF>BC1=BC2=BC3>BC4	1	24/25
	Entropy	**BC5**>NN>**SVM**>RF>BC1=BC2=BC3>BC4		

Table F. Results for the MONK's Problems. In boldface classifiers ranked differently with CEN and MCEN.

CEN and MCEN only give different rankings among the classifiers in problem 3, which is the only one with noise, and MCEN minimizes Hamming distance and maximizes the degree of consistency. The conclusion is the same as with the previous datasets.

References

1. R Core Team. R: A language and environment for statistical computing. R Foundation for Statistical Computing, Vienna, Austria (2018). https://www.R-project.org/

2. Scutari,M. Learning Bayesian Networks with the bnlearn R Package. Journal of Statistical Software, 35(3), 1-22 (2010). http://www.jstatsoft.org/v35/i03/

3. Højsgaard, S. Graphical Independence Networks with the gRain Package for R. Journal of Statistical Software, 46(10), 1-26 (2012). http://www.jstatsoft.org/v46/i10/

4. Grosjean, Ph., Denis, K. mlearning: Machine learning algorithms with unified interface and confusion matrices. R package version 1.0-0 (2013). https://CRAN.R-project.org/package=mlearning

5. Huang, J., Ling, C.: Using AUC and Accuracy in Evaluating Learning Algoritms. IEEE Transactions on Knowledge and Data Engineering, vol. 17, 299–310 (2005). DOI: 10.1109/TKDE.2005.50

6. Dua, D., Karra Taniskidou, E. UCI Machine Learning Repository [http://archive.ics.uci.edu/ml]. Irvine, CA: University of California, School of Information and Computer Science (2017).
