# Supplementary material for: Enhancing Confusion Entropy (CEN) for binary and multiclass classification
Source: PLoS One. 2019 Jan 14;14(1):e0210264. doi: 10.1371/journal.pone.0210264 (PMC6331113; doi:10.1371/journal.pone.0210264)
Supplement: S3 File — (PDF) [file pone.0210264.s003.pdf]

```
Citation Request:
   This breast cancer domain was obtained from the University Medical Centre,
   Institute of Oncology, Ljubljana, Yugoslavia.  Thanks go to M. Zwitter and
   M. Soklic for providing the data.  Please include this citation if you plan
   to use this database.

1. Title: Breast cancer data (Michalski has used this)

2. Sources:
   -- Matjaz Zwitter & Milan Soklic (physicians)
      Institute of Oncology
      University Medical Center
      Ljubljana, Yugoslavia
   -- Donors: Ming Tan and Jeff Schlimmer (Jeffrey.Schlimmer@a.gp.cs.cmu.edu)
   -- Date: 11 July 1988

3. Past Usage: (Several: here are some)
     -- Michalski,R.S., Mozetic,I., Hong,J., & Lavrac,N. (1986). The
        Multi-Purpose Incremental Learning System AQ15 and its Testing
        Application to Three Medical Domains.  In Proceedings of the
        Fifth National Conference on Artificial Intelligence, 1041-1045,
        Philadelphia, PA: Morgan Kaufmann.
        -- accuracy range: 66%-72%
     -- Clark,P. & Niblett,T. (1987). Induction in Noisy Domains.  In
        Progress in Machine Learning (from the Proceedings of the 2nd
        European Working Session on Learning), 11-30, Bled,
        Yugoslavia: Sigma Press.
        -- 8 test results given: 65%-72% accuracy range
     -- Tan, M., & Eshelman, L. (1988). Using weighted networks to
        represent classification knowledge in noisy domains.  Proceedings
        of the Fifth International Conference on Machine Learning, 121-134,
        Ann Arbor, MI.
        -- 4 systems tested: accuracy range was 68%-73.5%
    -- Cestnik,G., Konenenko,I, & Bratko,I. (1987). Assistant-86: A
       Knowledge-Elicitation Tool for Sophisticated Users.  In I.Bratko
       & N.Lavrac (Eds.) Progress in Machine Learning, 31-45, Sigma Press.
       -- Assistant-86: 78% accuracy

4. Relevant Information:
     This is one of three domains provided by the Oncology Institute
     that has repeatedly appeared in the machine learning literature.
     (See also lymphography and primary-tumor.)

     This data set includes 201 instances of one class and 85 instances of
     another class.  The instances are described by 9 attributes, some of
     which are linear and some are nominal.

5. Number of Instances: 286

6. Number of Attributes: 9 + the class attribute

7. Attribute Information:
   1. Class: no-recurrence-events, recurrence-events
   2. age: 10-19, 20-29, 30-39, 40-49, 50-59, 60-69, 70-79, 80-89, 90-99.
   3. menopause: lt40, ge40, premeno.
   4. tumor-size: 0-4, 5-9, 10-14, 15-19, 20-24, 25-29, 30-34, 35-39, 40-44,
                  45-49, 50-54, 55-59.
   5. inv-nodes: 0-2, 3-5, 6-8, 9-11, 12-14, 15-17, 18-20, 21-23, 24-26,
                 27-29, 30-32, 33-35, 36-39.
   6. node-caps: yes, no.
   7. deg-malig: 1, 2, 3.
   8. breast: left, right.
   9. breast-quad: left-up, left-low, right-up, right-low, central.
  10. irradiat: yes, no.

8. Missing Attribute Values: (denoted by "?")
   Attribute #:  Number of instances with missing values:
```

```
   6.            8
   9.            1.

9. Class Distribution:
    1. no-recurrence-events: 201 instances
    2. recurrence-events: 85 instances
```
