# Supplementary material for: Enhancing Confusion Entropy (CEN) for binary and multiclass classification
Source: PLoS One. 2019 Jan 14;14(1):e0210264. doi: 10.1371/journal.pone.0210264 (PMC6331113; doi:10.1371/journal.pone.0210264)
Supplement: S5 File — (PDF) [file pone.0210264.s005.pdf]

```
1. Title of Database: SPECT heart data

2. Sources:
   -- Original owners: Krzysztof J. Cios, Lukasz A. Kurgan
        University of Colorado at Denver, Denver, CO 80217, U.S.A.
        Krys.Cios@cudenver.edu
        Lucy S. Goodenday
        Medical College of Ohio, OH, U.S.A.

   -- Donors: Lukasz A.Kurgan, Krzysztof J. Cios
   -- Date: 10/01/01

3. Past Usage:
        1. Kurgan, L.A., Cios, K.J., Tadeusiewicz, R., Ogiela, M. & Goodenday, L.S.
           "Knowledge Discovery Approach to Automated Cardiac SPECT Diagnosis"
           Artificial Intelligence in Medicine, vol. 23:2, pp 149-169, Oct 2001

        Results: The CLIP3 machine learning algorithm achieved 84.0% accuracy
        References:
                Cios, K.J., Wedding, D.K. & Liu, N.
                CLIP3: cover learning using integer programming.
                Kybernetes, 26:4-5, pp 513-536, 1997

                Cios, K.J. & Kurgan, L.
                Hybrid Inductive Machine Learning: An Overview of CLIP Algorithms,
                In: Jain, L.C., and Kacprzyk, J. (Eds.)
                   New Learning Paradigms in Soft Computing,
                   Physica-Verlag (Springer), 2001

        SPECT is a good data set for testing ML algorithms; it has 267 instances
        that are descibed by 23 binary attributes

        Other results (in press):
                -- CLIP4 algorithm achieved 86.1% accuracy
                -- ensemble of CLIP4 classifiers achieved 90.4% accuracy
                -- Predicted attribute: OVERALL_DIAGNOSIS (binary)

4. Relevant Information:
        The dataset describes diagnosing of cardiac Single Proton Emission Computed
Tomography (SPECT) images.
        Each of the patients is classified into two categories: normal and
abnormal.
        The database of 267 SPECT image sets (patients) was processed to extract
features that summarize the original SPECT images.
        As a result, 44 continuous feature pattern was created for each patient.
        The pattern was further processed to obtain 22 binary feature patterns.
        The CLIP3 algorithm was used to generate classification rules from these
patterns.
        The CLIP3 algorithm generated rules that were 84.0% accurate (as compared
with cardilogists' diagnoses).

5. Number of Instances: 267
6. Number of Attributes: 23 (22 binary + 1 binary class)
7. Attribute Information:
   1.  OVERALL_DIAGNOSIS: 0,1 (class attribute, binary)
   2.  F1:  0,1 (the partial diagnosis 1, binary)
   3.  F2:  0,1 (the partial diagnosis 2, binary)
   4.  F3:  0,1 (the partial diagnosis 3, binary)
   5.  F4:  0,1 (the partial diagnosis 4, binary)
   6.  F5:  0,1 (the partial diagnosis 5, binary)
   7.  F6:  0,1 (the partial diagnosis 6, binary)
   8.  F7:  0,1 (the partial diagnosis 7, binary)
   9.  F8:  0,1 (the partial diagnosis 8, binary)
   10. F9:  0,1 (the partial diagnosis 9, binary)
   11. F10: 0,1 (the partial diagnosis 10, binary)
   12. F11: 0,1 (the partial diagnosis 11, binary)
   13. F12: 0,1 (the partial diagnosis 12, binary)
```

```
   14. F13: 0,1 (the partial diagnosis 13, binary)
   15. F14: 0,1 (the partial diagnosis 14, binary)
   16. F15: 0,1 (the partial diagnosis 15, binary)
   17. F16: 0,1 (the partial diagnosis 16, binary)
   18. F17: 0,1 (the partial diagnosis 17, binary)
   19. F18: 0,1 (the partial diagnosis 18, binary)
   20. F19: 0,1 (the partial diagnosis 19, binary)
   21. F20: 0,1 (the partial diagnosis 20, binary)
   22. F21: 0,1 (the partial diagnosis 21, binary)
   23. F22: 0,1 (the partial diagnosis 22, binary)
   -- dataset is divided into:
        -- training data ("SPECT.train" 80 instances)
        -- testing data ("SPECT.test" 187 instances)
8. Missing Attribute Values: None
9. Class Distribution:
   -- entire data
        Class           # examples
          0               55
          1               212
   -- training dataset
        Class           # examples
          0               40
          1               40
   -- testing dataset
        Class           # examples
          0               15
          1               172
```
