# Supplementary material for: Enhancing Confusion Entropy (CEN) for binary and multiclass classification
Source: PLoS One. 2019 Jan 14;14(1):e0210264. doi: 10.1371/journal.pone.0210264 (PMC6331113; doi:10.1371/journal.pone.0210264)
Supplement: S7 File — (PDF) [file pone.0210264.s007.pdf]

UCBAdmissions {datasets} R Documentation

Student Admissions at UC Berkeley

Description

Aggregate data on applicants to graduate school at Berkeley for the six largest departments in 1973 classified by admission and sex.

Usage

```
UCBAdmissions
```

Format

A 3-dimensional array resulting from cross-tabulating 4526 observations on 3 variables. The variables and their levels are as follows:

No	Name	Levels
1	Admit	Admitted, Rejected
2	Gender	Male, Female
3	Dept	A, B, C, D, E, F

Details

This data set is frequently used for illustrating Simpson's paradox, see Bickel *et al* (1975). At issue is whether the data show evidence of sex bias in admission practices. There were 2691 male applicants, of whom 1198 (44.5%) were admitted, compared with 1835 female applicants of whom 557 (30.4%) were admitted. This gives a sample odds ratio of 1.83, indicating that males were almost twice as likely to be admitted. In fact, graphical methods (as in the example below) or log-linear modelling show that the apparent association between admission and sex stems from differences in the tendency of males and females to apply to the individual departments (females used to apply *more* to departments with higher rejection rates).

This data set can also be used for illustrating methods for graphical display of categorical data, such as the general-purpose mosaicplot or the fourfoldplot for 2-by-2-by-*k* tables.

References

Bickel, P. J., Hammel, E. A., and O'Connell, J. W. (1975). Sex bias in graduate admissions: Data from Berkeley. *Science*, **187**, 398–403. http://www.jstor.org/stable/1739581.

Examples

```
require(graphics)
## Data aggregated over departments
apply(UCBAdmissions, c(1, 2), sum)
mosaicplot(apply(UCBAdmissions, c(1, 2), sum),
           main = "Student admissions at UC Berkeley")
## Data for individual departments
opar <- par(mfrow = c(2, 3), oma = c(0, 0, 2, 0))
for(i in 1:6)
  mosaicplot(UCBAdmissions[,,i],
    xlab = "Admit", ylab = "Sex",
    main = paste("Department", LETTERS[i]))
mtext(expression(bold("Student admissions at UC Berkeley")),
```

```
      outer = TRUE, cex = 1.5)
par(opar)
```

[Package *datasets* version 3.6.0 Index]
